# Interaction of Pestiviral E1 and E2 Sequences in Dimer Formation and Intracellular Retention

**DOI:** 10.3390/ijms22147285

**Published:** 2021-07-06

**Authors:** Yu Mu, Birke Andrea Tews, Christine Luttermann, Gregor Meyers

**Affiliations:** 1 Institut für Immunologie, Friedrich-Loeffler-Institut, D-17493 Greifswald-Insel Riems, Germany; muyu19900919@163.com (Y.M.); christine.luttermann@fli.de (C.L.); 2 Institut für Infektionsmedizin, Friedrich-Loeffler-Institut, D-17493 Greifswald-Insel Riems, Germany; birke.tews@fli.de

**Keywords:** pestivirus, RNA virus envelope protein, envelope protein heterodimer, envelope protein homooligomer, intracellular retention, intracellular localization

## Abstract

Pestiviruses contain three envelope proteins: E^rns^, E1, and E2. Expression of HA-tagged E1 or mutants thereof showed that E1 forms homodimers and -trimers. C123 and, to a lesser extent, C171, affected the oligomerization of E1 with a double mutant C123S/C171S preventing oligomerization completely. E1 also establishes disulfide linked heterodimers with E2, which are crucial for the recovery of infectious viruses. Co-expression analyses with the HA-tagged E1 wt/E1 mutants and E2 wt/E2 mutants demonstrated that C123 in E1 and C295 in E2 are the critical sites for E1/E2 heterodimer formation. Introduction of mutations preventing E1/E2 heterodimer formation into the full-length infectious clone of BVDV CP7 prevented the recovery of infectious viruses, proving that C123 in E1 and C295 in E2 play an essential role in the BVDV life cycle, and further support the conclusion that heterodimer formation is the crucial step. Interestingly, we found that the retention signal of E1 is mandatory for intracellular localization of the heterodimer, so that absence of the E1 retention signal directs the heterodimer to the cell surface even though the E2 retention signal is still present. The covalent linkage between E1 and E2 plays an essential role for this process.

## 1. Introduction

Pestiviruses are classified as members of the family *Flaviviridae*, and are among the most important pathogens of farm animals worldwide [1]. The members of the genus *Pestivirus* were originally believed to only infect pigs and ruminants where they induce a variety of clinical manifestations in farm or wild animals. Even though several good vaccines against the most important pestiviruses have been developed and a series of strict bio-safety measures like quarantine and stamping-out strategies have long been carried out, pestiviruses cause severe financial losses in the animal farming industry [2,3,4,5]. More recent studies revealed the existence of a variety of exotic pestiviruses with a much broader host range, leading to a new classification scheme [6,7].

Three envelope proteins, E^rns^, E1, and E2, are present on the pestiviral particle [8,9]. E1 is by far the least characterized component of the virion with neither structure nor function analyzed in detail so far. The molecular size of glycosylated E1 is 27–33 kDa, depending on the virus species. This is only about half the size of E2. The glycoprotein E1 of the closely related HCV was shown to form non-covalently linked trimers on the virion, which are of functional importance [10]. Both E^rns^ and E2 of pestiviruses can form homodimers that are found in infected cells and virions [8,11,12,13]. Because of the absence of robustly reacting specific antibodies against E1, it is still unknown whether E1 of pestiviruses forms oligomers or not. 

E1 forms disulfide linked heterodimers with E2 [8], and this structure was suggested to be important for pestivirus infection since absence of heterodimers prevented infectivity of vesicular stomatitis viruses pseudotyped with bovine viral diarrhea virus (BVDV) envelope proteins E1 and E2 [14]. This publication reported that two positively charged residues in the E1 membrane anchor play a role in heterodimer formation since replacement of these residues by alanine reduced the amount of heterodimer. In addition, the cysteine residue at position 668 in the polyprotein (residue 171 in E1) was claimed to be not essential [14]. Similarly, for HCV, it has been shown that the charged residues within the transmembrane domains of glycoproteins E1 and E2 play an important role in E1/E2 heterodimerization [15,16]. However, the interaction between HCV E1 and E2 in infected cells is non-covalent, and, therefore, the interaction mechanism of HCV E1 and E2 can be hypothesized to be different from that of pestiviruses. So far, experimental data are missing that could reveal which cysteines of E1 and E2 of pestiviruses play essential roles in E1/E2 heterodimer formation. Crystal structure investigation of BVDV E2 showed that, except for the cysteine residue at position 295 in E2, all the other cysteines of E2 formed intramolecular disulfide bonds [17,18]. Accordingly, C295 is the only free cysteine residue in E2, which makes this site the logical candidate for the necessary disulfide linkage in dimers. Convincing experimental evidence is also missing that could clarify which of the E1 cysteines is involved in E1/E2 heterodimer formation. It was suggested that C171 in E1 forms a disulfide bond with C295 in E2, based on the results of computational secondary structure predictions and E1/E2 sequence alignments [19], along with the geometric constraints imposed by the recently published crystal structure of BVDV E2 [17,18]. In addition, other cysteine residues in E1 have been proposed to be engaged in heterodimer formation with E2 [20].

It is strongly suggested that amino acids important for the interaction of E1 and E2 and perhaps the formation of the heterodimers itself could also have an influence on intracellular retention of the envelope proteins. The arginine at position 355 in E2 has a large effect on the retention of E2 since the mutation R355A resulted in the expression of the protein on the cell surface [21]. Work in our lab demonstrated that other changes like R355K, R355Δ, and Q370E also had effects on the cell surface presence of E2 with varying degrees [22]. R355 is not only important for the retention but is also very important for heterodimer formation, which is completely abolished in E2 R355A mutants [14]. Interestingly, the published data also showed that co-expression of E1 can compensate for the reduced retention of E2 R355K and E2 Q370E, whereas it had no/very slight effects on the other two retention-defective E2 mutants R355A and R355Δ [22]. These data suggest that E1 has the dominant retention signal and that it can still interact with E2 R355K and E2 Q370 and thus rescue the E2 phenotype. Viruses carrying these mutations were also viable [22]. Moreover, we recently demonstrated that E1 indeed contains an independent retention signal [23]. Nevertheless, this leaves us with an incomplete understanding of the requirements in E1 for heterodimerization. Due to the absence of experimental structural data for E1 or the E1/E2 heterodimer, the prerequisites and function of E1 oligomerization and E1–E2 heterodimerization are poorly understood. Here, we analyzed pestiviral E1 oligomerization and heterodimerization with E2 in detail and further investigated the importance of these processes for the recovery of infectious viruses. We identify the cysteine residues involved in the formation of covalently linked oligomers and demonstrate that E1/E2 heterodimerization is crucial for the recovery of infectious pestiviruses.

## 2. Results

### 2.1. Pestiviral Glycoprotein E1 Can Form Homooligomers Independent of Its Membrane Anchor

**Figure 1:**
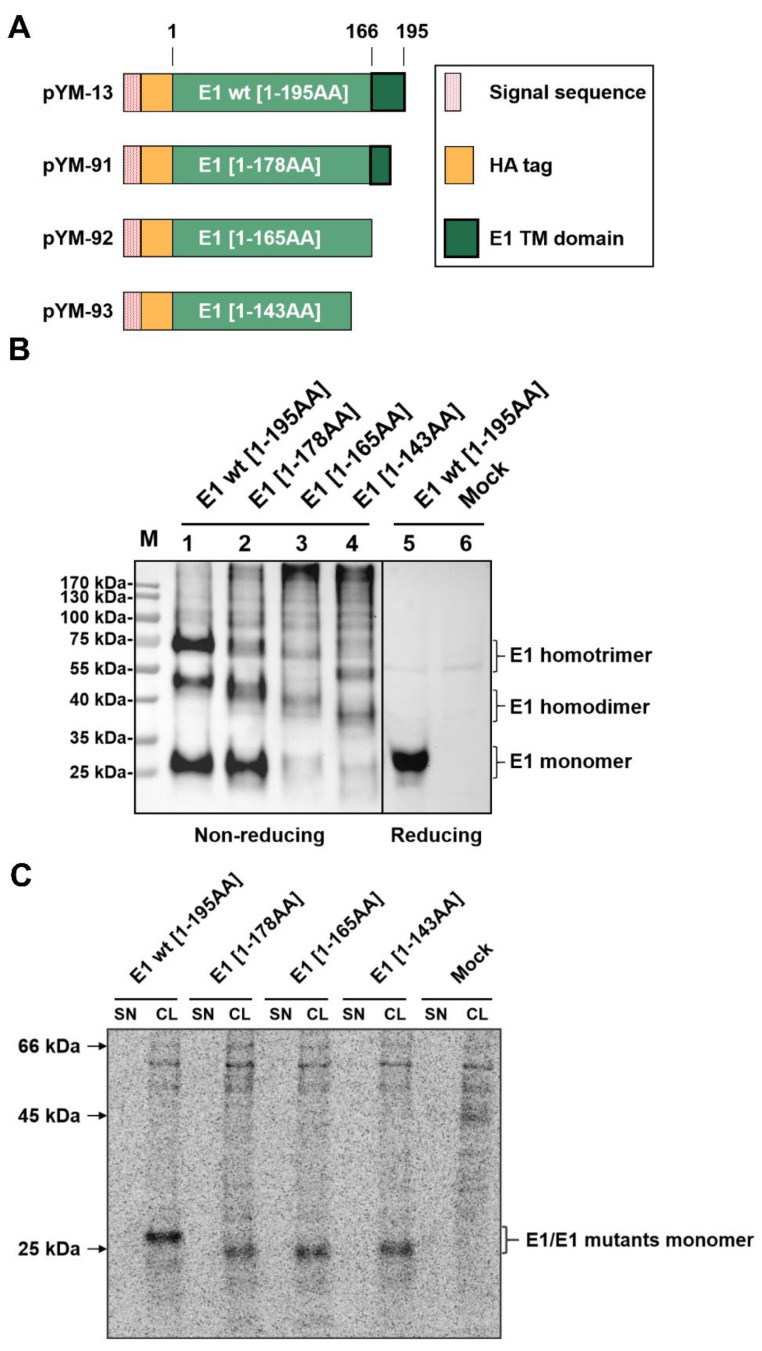
E1 forms homooligomers independent to its membrane anchor. (**A**) Schematic representation of HA-tagged E1 wt/C-terminally truncated E1 proteins expressed from the constructs. The box on the right gives the meaning of the differently designed bars [red dotted bar: E^rns^ signal sequence fused to the E1 sequences (green bars) with the length of the sequences given]. A HA-tag (orange bar) was introduced in between the signal sequence and the E1 sequence for the detection of E1. The black frame represents the putative transmembrane region of E1. (**B**) The HA-tagged E1 and its truncated variants were expressed in RK-13 cells, and expression products were analyzed via western blot on the following day. The cells were lysed with lysis buffer without (**left**) or with (**right**) β-mercaptoethanol, aliquots of the lysates loaded onto SDS gels and separated electrophoretically. Western blot analysis was carried out using primary antibody α-HA and PO-labeled second antibodies for the detection of protein. For each lane, the length of the expressed E1 sequence is given on top. Molecular weight of the size marker bands given on the left. As a supplement, we provide a file with the original western blot results to demonstrate that the two parts were derived from one gel. (**C**) The HA-tagged E1 expression plasmids were expressed in BHK-21 cells using the Vaccinia virus MVA T7 expression system [23,30,31], and the empty pCI empty vector served as a mock control. Expressed proteins were labelled with ^35^S amino acids. From supernatant (SN) and cell lysates (CL) of the transfected cells, proteins were precipitated with a specific HA-tag antiserum, separated by SDS-PAGE under reducing conditions detected on imaging plates. Molecular weight of the size of the marker bands given on the left.

The glycoproteins E1 and E2 of HCV were shown to form non-covalently linked trimers or dimers on the virion, respectively [10,24,25,26,27,28,29]. The envelope proteins E^rns^ and E2 of pestiviruses can both form homodimers [8,11,12,13]. In addition, pestiviral E2 was also identified as a component of a covalently linked heterodimer with E1 [8]. Because of the absence of robustly reacting specific antibodies against E1, it is still unknown whether E1 of pestiviruses forms homooligomers or not. Therefore, we expressed E1 carrying a N-terminal HA-tag (construct pYM-13, Figure 1A and [23]) and analyzed the expression products under non-reducing conditions. Upon transient expression and western blot analysis, three predominant bands were detected by SDS-PAGE under nonreducing conditions (Figure 1B, left part). Since the same procedure but with electrophoresis under reducing conditions yielded only one band (Figure 1B, right part) and the electrophoretic mobility of the higher molecular weight bands corresponded to homotrimer and homodimer of E1, the results strongly argue in favor of the homooligomerization of E1. A ladder of weak specific bands of higher MW was detected that might even represent oligomers composed of higher numbers of E1 molecules. Thus, we observed for the first time that pestiviral E1 can form homooligomers. 

It was shown for HCV that the membrane anchors of E1 and E2 are crucial for oligomerization. The same is true for pestiviral E2, where R355 also lies in the membrane anchor. To determine whether the C-terminal membrane anchor is also important for the oligomerization of pestiviral E1, we constructed a series of E1 mutants with truncations of different length at the Carboxy-terminus and tested them via western blot (Figure 1A,B). Surprisingly, the E1 mutants with truncations deleting the membrane anchor region (pYM-92 and pYM-93) still yielded three clear bands in the transfected cell lysates, migrating a bit faster than the HA-E1 wt bands due to the decreased molecular weights (Figure 1B). This result indicated that pestivirus E1 can form homooligomers even in the absence of the Carboxy-terminal transmembrane region. It is worth noting that the signal from the proteins detected in pYM-91 transfected cells was still comparable to that of the HA-E1 wt. However, weaker bands were observed for the truncated samples with longer deletions (pYM-92 and pYM-93), which might be an indication for the secretion of those truncated E1 proteins lacking the membrane anchor. To test for this, we employed immunoprecipitation analyses with lysates and supernatants of cells transiently expressing the different versions of E1. We used this technique because larger amounts of the supernatant can be included in the test, which together with radioactive labelling of the proteins increases sensitivity. However, secretion of the truncated polypeptides was not detectable (Figure 1C). For E1 [1-143] (construct pYM-93), a low amount of secreted protein could be observed after prolonged exposure time [23], whereas the C-terminally truncated E1 lacking residues 179 to 195 or 166 to 195 (constructs pYM91 or pYM-92, respectively) was not detectable at all in our experimental system (Figure 1C). The lower amounts detected for constructs pYM-92 and pYM-93 might therefore result from instability of the truncated proteins.

### 2.2. Both C123 and C171 Play an Important Role in E1 Homooligomerization

**Figure 2:**
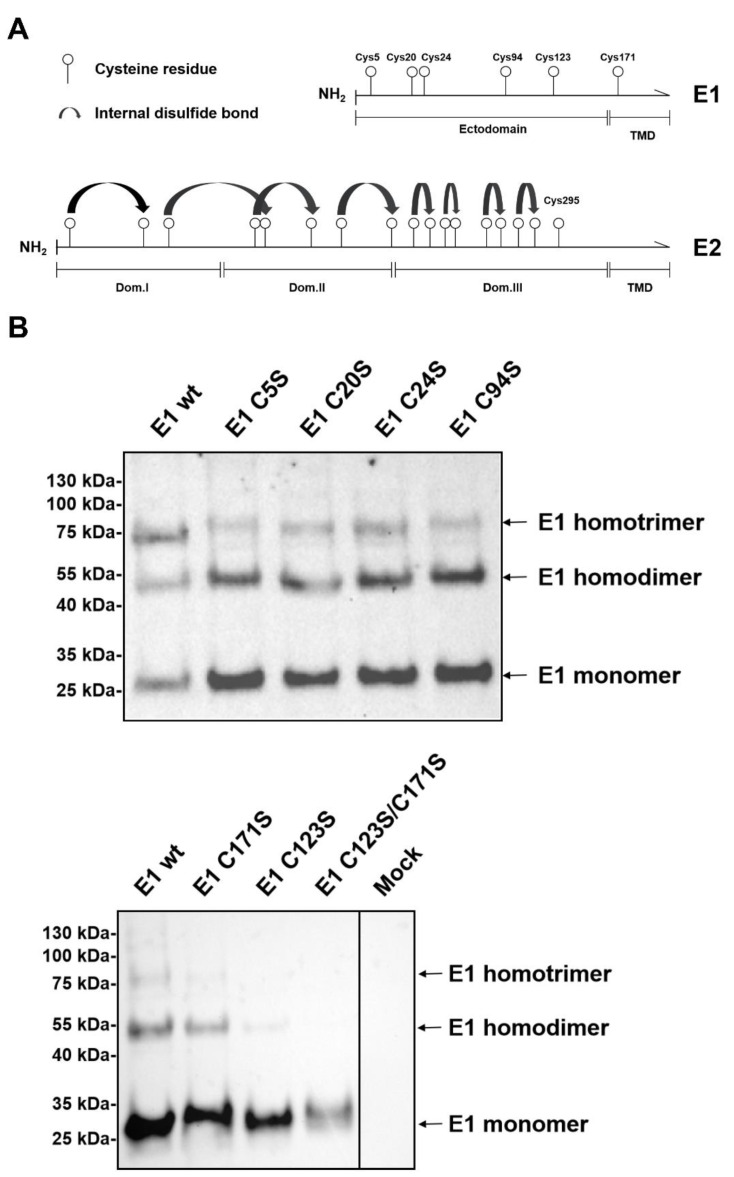
Both C123 and C171 play an important role in E1 homooligomerization. (**A**) Schematic representation of the distribution of cysteine residues in the E1 and E2 proteins of pestiviruses (BVDV CP7 as an example). Numbered circles represent the positions of the Cys residues in E1 and E2. Curved black arrows indicate internal disulfide bonds identified during E2 3D structure determination [17,18]. (**B**) The given constructs coding for HA-tagged E1 wt and mutants with Cys to Ser exchanges were transfected into RK-13 cells and expression products were analyzed via western blot. The cells were lysed and separated by SDS-PAGE under nonreducing conditions. Western blot analysis was carried out using primary antibody α-HA and secondary antibody anti-rabbit-PO for the detection of proteins. A file is provided in the Supplementary Materials with the original western blot results to demonstrate that the two parts of the lower panel were derived from one gel.

Pestiviral envelope proteins E^rns^ and E2 generate homodimers via intermolecular disulfide bonds established by their cysteine residues closest to the C-terminus [11,17,18]. The homooligomerization of E1 is also mediated by disulfide bonds since SDS-PAGE under reducing conditions eliminates detection of oligomers and shows only the E1 monomeric band (Figure 1). There are six cysteine residues in E1 of BVDV CP7 (Figure 2A). According to the alignment shown in the Supplementary Materials, the first five cysteine residues (at position 5, 20, 24, 94, and 123) are highly conserved throughout nearly all pestivirus species and can therefore be considered as important for the stability and function of E1. Based on computational model predictions, it was suggested that C171 should be the critical site for E1/E2 heterodimer formation [19]. However, in BVDV-2 strains, the sixth cysteine at position 171 is missing (S1), indicating that Cys at this site could be dispensable. To obtain experimental evidence for the identification of the cysteine residue(s) engaged in disulfide linkage of E1 oligomers, a series of E1 mutants containing single or double exchanges (Cys to Ser) were generated by site-directed mutagenesis. HA-tagged E1 wt and these mutants were expressed in RK-13 cells, and separated by SDS-PAGE under non-reducing conditions, and detected by Western blot with anti-HA serum. Compared to HA-E1 wt, the individual substitution of one of the first four cysteine residues had no significant effect on E1 oligomerization (Figure 2B, mutants C5S, C20S, C24S, and C94S). Interestingly, both C123S and C171S reduced the presence of homotrimer and homodimer of E1, with the exchange of C123 having a stronger influence on the oligomer formation of E1. If C123S/C171S double mutations were introduced into E1, only the E1 monomer could be detected on the blot (Figure 2B). Even after prolonged exposure, no dimer band became visible. These data indicated that the first four cysteine residues most likely form intramolecular disulfide bonds, while the last two cysteines are involved in intermolecular linkage. In this context, C123 is obviously more important than C171.

### 2.3. Critical Sites for E1/E2 Heterodimer Formation and E2 Homodimer Formation 

**Figure 3:**
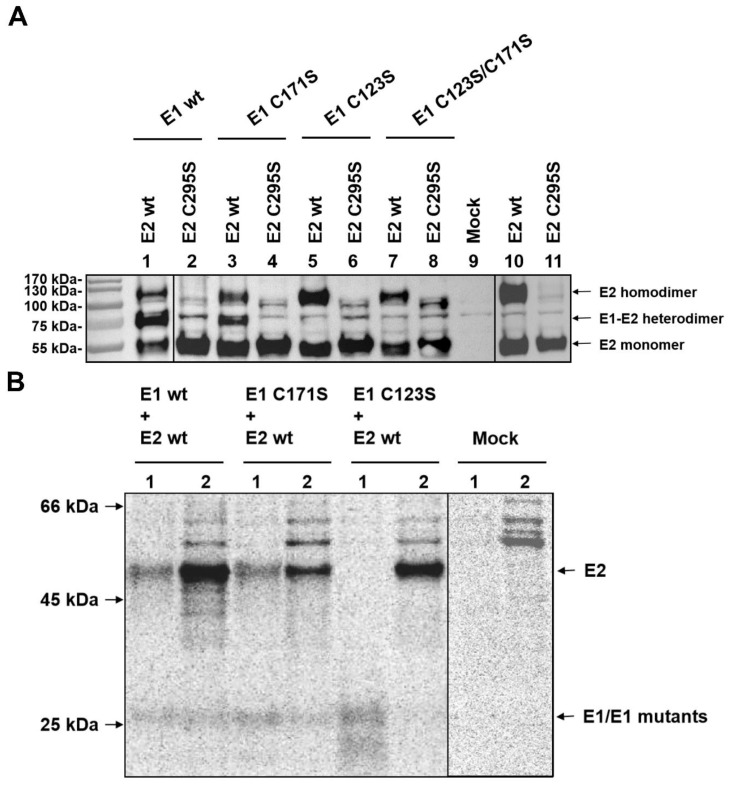
C123 in E1 is the critical residue for E1/E2 heterodimer formation; C295 in E2 is essential for not only E1/E2 heterodimerization but also for E2 homodimer formation. (**A**) The given HA-tagged E1 wt or cysteine lacking mutants were co-expressed with AU1-tagged E2 or E2 C295S mutant in RK-13 cells, and expression products were analyzed via Western blot on the following day. Cell lysates were prepared and separated by SDS-PAGE under nonreducing conditions. Western blot analysis was carried out using primary antibody α-AU1 and secondary antibody anti-rabbit-PO for the detection of protein. Above the gel, the composition of the samples is given. (**B**) E1 mutants containing single mutations (Cys to Ser) were also tested in this study. The E1 mutants C171S and C123S were co-expressed with E2 wt in RK-13 cells and analyzed via immunoprecipitation with either HA antiserum (lanes 1) or AU1 antiserum (lanes 2). The combinations of constructs transfected into the cells are given above the gel. A file is provided in the Supplementary Materials with the original Western blot results to demonstrate that the different parts presented here were derived from equivalent gels.

Mutations in the Carboxy-terminal part of E2 that prevent establishment of covalently disulfide-linked E1/E2 heterodimers were shown to interfere with infectivity of pseudotyped vesicular stomatitis virus (VSV) so that heterodimer formation could also be needed for pestiviral infectivity [14]. Published data [17,18] suggest that except for the cysteine residue at position 295 in E2, the remaining cysteines of E2 formed intramolecular disulfide bonds. Thus, C295 is the only free cysteine residue in E2, which renders this site the prime candidate for the disulfide linkage in homodimers or the heterodimer with E1. However, in light of our results described above and published in silico analyses, it is unclear which of the E1 cysteines is involved in E1/E2 heterodimer formation. To identify the responsible residue in E1 for E1/E2 crosslinking, AU1-tagged E2 or E2 mutants were co-expressed with HA-tagged E1 or mutants thereof. A substitution of C295S was introduced into E2 for blocking the only unlinked cysteine residue in E2. HA-E1 wt or HA-E1 mutants containing single and double mutations (Cys to Ser) were co-transfected with AU1-E2 wt or AU1-E2 mutant C295S in the RK-13 cells, respectively, and the presence of E1/E2 heterodimers was analyzed by western blotting with an anti-AU1 serum after electrophoretic separation of the cellular proteins under non-reducing conditions. Co-expression of HA-E1 wt with AU1-E2 wt (Figure 3A, lane 1) resulted in a dominant band of ~75 kDa, which according to size corresponds to the E1/E2 heterodimer. In addition, bands of ~55 and ~110 kDa were visible that represent the E2 monomer and homodimer, respectively. However, when HA-E1 wt was co-expressed with AU1-E2 C295S, the E1/E2 heterodimer band was no longer visible and the E2 homodimer band was diminished dramatically to a level that made it difficult to decide whether it represents a specific signal (Figure 3A, lane 2). This situation was also true for other E1 variants when co-expressed with AU1-E2 C295S (Figure 3A, lanes 4, 6, and 8). 

When AU1-E2 wt was expressed alone (Figure 3A, lane 10), we could see the E2 homodimer together with similar amounts of the E2 monomer. In contrast, when the only free cysteine in E2 was blocked, the E2 homodimer was reduced to a similarly low level as observed for the co-expression of this mutant with HA-E1 (Figure 3A, lanes 11 and 2, respectively). These results are in agreement with the structure analysis reports suggesting that the cysteine residue at position 295 in E2 is critical for both E1/E2 heterodimerization and E2 homodimerization [17,18,19].

To further verify the conclusions from the western blot experiment, we performed immunoprecipitation after co-expression of HA-tagged E1 wt/E1 mutants and AU1-tagged E2 wt, and subsequently separated the precipitates under reducing conditions. This method was chosen because, for unknown reasons, detection of the HA-E1/AU1-E2 heterodimer with the anti HA-sera we tested did not work in WB. As shown in Figure 3B, when HA-E1 wt was co-expressed with AU1-E2 wt, E2 can be precipitated together with E1 from denatured protein lysates with specific antibodies against the HA-tag, which proved the presence of a disulfide linkage between the two proteins. E1 was also detected when the AU1-tag antibody directed against AU1-E2 was used for precipitation (Figure 3B, left two lanes). Similarly, E1 (C171S) can still be precipitated with antibodies against both the HA-tag and the AU1-tag, and, *vice versa*, co-precipitation of E2 was observed when HA-E1 was captured with anti-HA antibodies (Figure 3B, lanes 3 and 4 from the left). These findings indicate that C171 in E1 is dispensable in E1/E2 heterodimer formation. When HA-E1 (C123S) was co-expressed with AU1-E2 wt, only one dominant band representing E1 or E2 were visible in the corresponding lanes, but no co-precipitation of the second partner, suggesting that stable interaction between E1 and E2 was not existing (Figure 3B, lanes 5 and 6 from the left). 

These results clearly show that C123 is the important site for E1/E2 heterodimer formation. This conclusion is contradictory to the computational prediction [19]. Moreover, our results also suggested that in the presence of E1, E2 prefers to form E1/E2 heterodimers instead of homodimers since the amount of E2 homodimers seemed to be lower in all cases where heterodimers were detected (comparison of lanes 1/3 with lanes 5/7/9 in Figure 3A). Thus, the E2 homodimer might be an excess product for E2 molecules not engaged in E1/E2 heterodimer formation. 

### 2.4. E1/E2 Heterodimer Formation Is Independent of the Transmembrane Region of E1

**Figure 4:**
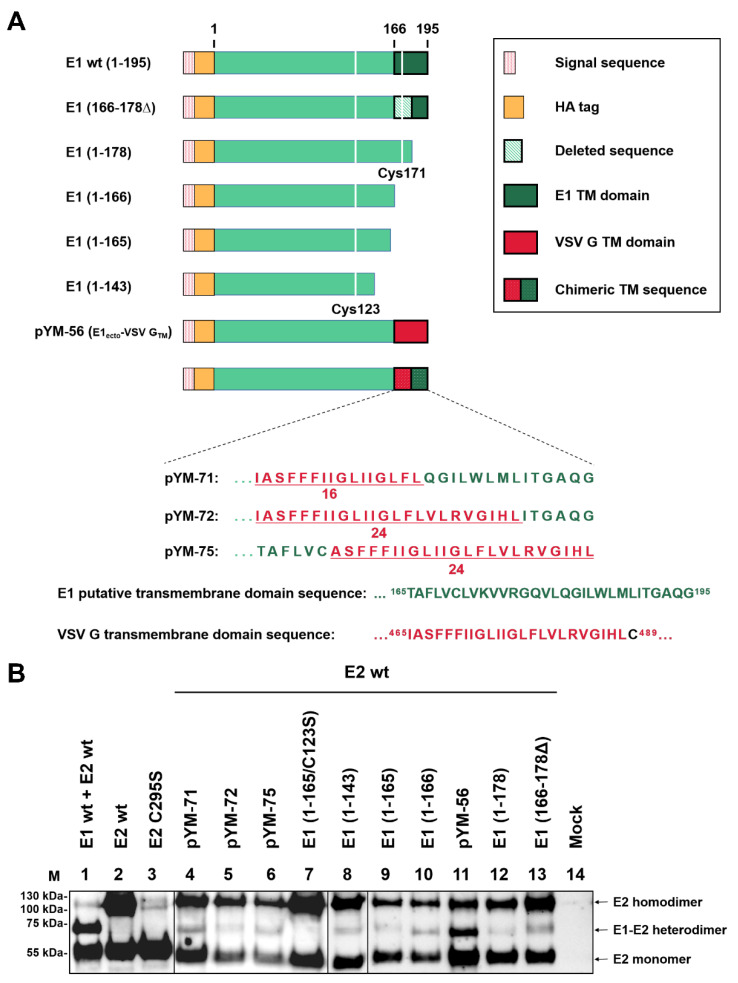
Heterodimer formation analysis. (**A**) Schematic representation of the HA-E1 wild type and Carboxy-terminally truncated E1 constructs used in this section. Cysteine residues C123 and C171 are presented as white lines in the green bar representing the E1 sequence. Further constructs shown here encode HA-E1 with the TM domain or parts thereof replaced by sequences from the TMD of VSV. Below the schemes, the amino acid sequences of the chimeric TMDs are given in the one letter code (see also [23]). (**B**) The given Carboxy-terminally truncated E1 expression plasmids were co-transfected with a plasmid coding for E2 wt into RK-13 cells. Co-expression products were analyzed via nonreducing SDS-PAGE and Western blot using mAb α-E2 (WB214) for the detection of the E1/E2 heterodimer and E2 monomer/homodimer. A file is provided in the Supplementary Materials with the original Western blot results to demonstrate that the two parts were derived from equivalent gels and the mock control was run on one of these gels.

We have shown that there is a dynamic membrane topology change of the transmembrane region of E1 after signal sequence cleavage with a relocation of the E1 Carboxy-terminus from the ER to the cytosolic side [23]. This is also true for E2 of pestiviruses [22] and the closely related HCV envelope proteins [32]. It is proposed that the relocation is linked to multifunctionality of the membrane anchor, which in both pesti- and hepaciviruses is responsible for anchoring as well as ER retention. Moreover, in hepaciviruses, the TMDs are also crucial for E1/E2 heterodimer formation. To determine whether the TMD of E1 also affects E1/E2 heterodimerization in pestiviruses, the Carboxy-terminally truncated E1 variants shown in Figure 4A were tested for their ability to form heterodimers. As already shown above, controls with co-expression of HA-E1 wt with E2 wt resulted predominantly in E1/E2 heterodimers (Figure 4B, lane 1), whereas E2 formed large amounts of E2 homodimers in the absence of E1 (lane 2). In contrast, homodimers were absent for the C295S mutant of E2 (lane 3). Different truncated variants of E1 were first analyzed in this study together with E2. Western blot showed that all the C-terminally truncated versions of E1 still formed E1/E2 heterodimers in the presence of wild type E2 (Figure 4B, lanes 8–10, 12,13). This is even also true for the constructs, in which the entire TMD was deleted as in construct E1 [1–143] (Figure 4B, lanes 8). However, the amount of heterodimers detected for all of the truncated versions of E1 was dramatically reduced. The truncated proteins were not secreted to a significant extent (Figure 1). Moreover, we did not see indications for instability as a consequence of truncation since the amounts of the truncated proteins detected in cells expressing only truncated E1 were not significantly lower than those of the control cells with HA-E1 wt. Only for the shortest version E1 [1–143] did we detect a decreased amount of protein (not shown). Thus, our results indicate that the E1 C-terminus with the membrane anchor is not crucial, but plays an important role for the efficiency of heterodimer formation (Figure 4B, lanes 8–10, 12, 13). 

To further analyze whether the membrane anchor function of the E1 TMD or the specific sequence of this element is important for dimerization, we examined the effect of the replacement of the TM domain of E1. Four representative constructs, in which the TMD of E1 was fully or partially replaced by that of VSV G, were tested. Interestingly, even when the TM region of E1 was fully or partially exchanged by the corresponding sequence of VSV G, E1/E2 heterodimers could be detected (Figure 4B, lanes 4–6, 11), but again, only low amounts were present. For these constructs, the reduced amount of heterodimer could be due to the fact that these chimeric proteins are transported to the cell surface in contrast to E1 wt [23]. In any case, it is obvious that the TM domain of E1 is not necessary for the dimerization of E1 and E2 in general, but renders it much more efficient. Dimerization in the absence of the TM domain is dependent on the presence of C123 since no heterodimer is detected for a E1 (1–165) C123S mutant (comparison of lanes 9 and 7 of Figure 4B). Notably, this shows that the heterodimers formed by truncated E1 rely on the same principles as those containing full length E1, and it also further proves that C123, not C171, is important for the heterodimer formation of E1 and E2.

### 2.5. E1 Is Dominant for the Retention of E1/E2 Heterodimers

Earlier work demonstrated that the arginine at position 355 in E2 has a large effect on the retention of E2 [21,22]. Mutations affecting R355 like R355Δ, R355A, R355K, and R355E, all resulted in cell surface exposure of E2. Among them, the former two had the strongest effects on the retention of E2 whereas the substitutions R355K and R355E had much weaker effects. Furthermore, these analyses showed that in E1/E2 heterodimers generated upon co-expression of E1 and E2, E1 can compensate for the reduced retention of some selected E2 mutants like R355K. The retention deficit of E2 mutants R355A and R355Δ, however, could not be compensated by E1 [22]. Ronecker and co-workers showed that the E2 R355A mutation prevents generation of E1/E2 heterodimers [14]. These results suggest that heterodimerization between E1 and E2 is essential for compensation of the reduced retention of E2 R355 mutants, arguing that E2 R355K probably still interacts with E1. R355 is very conserved among pestiviruses, but the existence of a few selected strains containing K355 further strengthens this point. 

**Figure 5:**
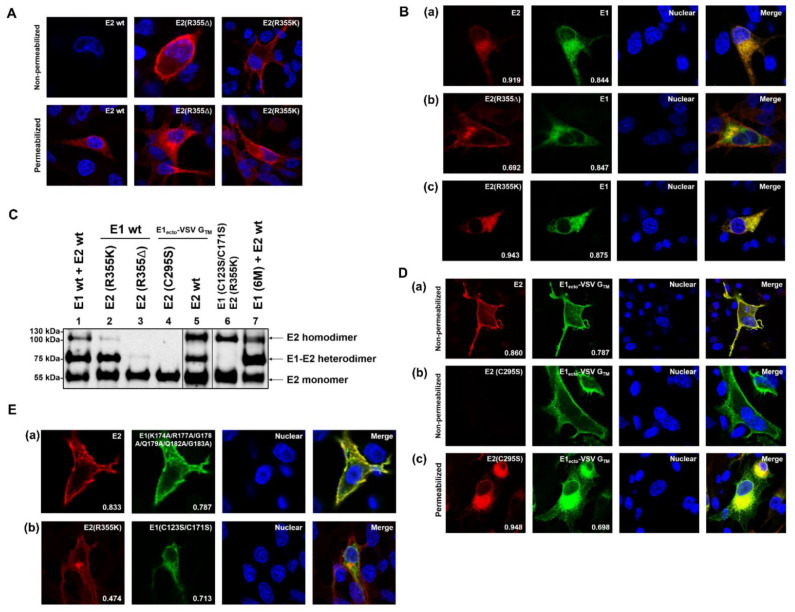
Presence or absence of the E1 retention signal determines the cellular localization of the E1/E2 heterodimer. (**A**) Subcellular localization analysis of E2 (or variants). BHK-21 cells were transfected with the given expression plasmids, fixed with 4% PFA on the following day and analyzed via confocal microscopy. E2 was detected with α-AU1 and α-abbit-AlexaFluor-594. (**B**,**D**,**E**) Co-localization analysis of E1 (or variants) and E2 (or variants), Mander’s coefficients for colocalization of E2 with E1, and E1 with E2 signal are displayed at the bottom of the pictures. Magnification 500×. BHK-21 cells were transfected with the given expression plasmids, fixed with 4% PFA on the following day and analyzed via confocal microscopy. In the lower row of A and (**D**(**c**)) all of the cell membranes were permeabilized with 0.05% Triton-X100 whereas in the upper row of A as well as in (**D**(**a**,**b**)), the samples were not permeabilized to analyze surface localization. Cells in (**B**,**E**) were all permeabilized with Triton-X100. E1 was detected with α-HA (IgG3), α-mouse (IgG3), and AlexaFluor-488; E2 was detected with α-E2 1b31 mAb (IgG1) from BVDV-Mix [33] and α-mouse (IgG1)-Cy3. (**C**) Western blot analysis for E1/E2 heterodimer formation of the indicated proteins. The given E1 wt/E1 mutant expression plasmids were co-transfected with plasmid coding for E2 wt/E2 mutants into RK-13 cells, respectively, and co-expression products were analyzed via nonreducing SDS-PAGE and western blot on the following day. Western blot analysis was carried out using primary antibody: mAb α-E2 (WB214), secondary antibody: α-mouse PO to show the E1/E2 heterodimer and E2 monomer/homodimer. A file is provided in the Supplementary Materials with the original western blot results to demonstrate that the different parts were derived from equivalent gels and that one of these gels contained a mock control.

To investigate the mechanism of this interesting phenomenon, we tested the cellular localization of wild type E1 and E2 or E2 mutants. Two representative E2 retention deficient mutants encoded by constructs pCR-78 (E2 R355K) and pCR-79 (E2 R355Δ) [22] were used in this study. The expression plasmid coding for HA-tagged E1 was co-transfected with plasmids coding for E2 or E2 mutants into BHK-21 cells. On the following day, the cells were fixed with 4% PFA and permeabilized with 0.05% Triton-X100. Staining was done with HA-tag antiserum plus Alexa Fluor 488 α-rabbit and BVDV E2 mAb Mix plus Alexa Fluor 594 α-mouse. As shown in Figure 5A, a specific signal presenting on the cell surface was not detected when E2 wt was expressed alone, since E2 contains the retention signal of its own [22]. Furthermore, when E2 wt was co-expressed with HA-E1 wt, both proteins showed a clear intracellular co-localization (Mcc E2 = 0.919, Mcc E1 = 0.844) without any cell surface localization (Figure 5B(a)). Compared to the co-localization observed upon co-expression of HA-E1 wt with E2 wt (Figure 5B(a)), the combination HA-E1 wt with E2 R355Δ gave significantly different results. While E1 was still retained within the cells and intracellular localization of E1 was still colocalized with intracellular distribution of E2 R355Δ (Mcc E1 = 0.847), a considerable part of E2 R355Δ was transported to the cell surface and no longer colocalized with E1 (Mcc E2 = 0.692) (Figure 5B(b)). Intracellular colocalization is probably at least in part due to the transport on the secretory pathway that E2 still has to take. Interestingly, wt E1 compensated the reduced E2 retention when R355 in E2 was replaced by lysine (R355K), resulting in a retention behavior similar to E2 wt (Mcc E2 = 0.943, Mcc E1 = 0.875) (Figure 5B(c)). These results agree with the earlier published confocal imaging and FACS data [22]. 

As a next step, we examined these two E2 mutants for their ability to form heterodimers with E1 wt. Electrophoretic separation of the proteins under non-reducing conditions followed by western blot was carried out. As shown in Figure 5C, E2 containing the mutation R355K could still form E1/E2 heterodimers. However, when the arginine at position 355 was deleted, E1/E2 heterodimers could not be detected at all. Moreover, the E2 homodimer was also no longer detectable (Figure 5C, lanes 1, 2, and 4). 

Furthermore, the representative construct pYM-56 (coding for HA-tagged E1_ecto_ VSV G _TMD_) was tested in this study. The protein expressed from this plasmid was shown previously to be exposed on the cell surface [23]. Since E2 wild type contains a retention signal of its own, we wanted to know whether E2 can keep a retention deficient E1 mutant within the cell via formation of heterodimers. For this purpose, pCR-16 coding for AU1-tagged BVDV CP7 E2 was co-expressed with pYM-56 in BHK-21 cells. Surprisingly, both the E1 retention deficient mutant and E2 were present on the cell surface (Figure 5D(a)) and showed a clear colocalization (Mcc E2 = 0.860, Mcc E1 = 0.787). This indicates that E1 can overrule the retention signal of E2. Western blot results showed that the retention deficient E1 mutant still formed heterodimers with E2 (Figure 5C, lane 3). However, when C295S was introduced into E2 to prevent the covalent linkage between E1 and E2 (Figure 5C, lane 5), E2 did not follow the E1 mutant to the cell surface. E2 colocalized with part of the intracellular E1 signal (Mcc E2 = 0.948) (Figure 5D(c)), but E1_ecto_-VSV-G_TDM_ only partially colocalized with E2 (Mcc E1 = 0.698). This was similar to the situation of E1 and E2 R355Δ, probably also due to E1 traversing the secretory pathway (Mcc E2 = 0.948, Mcc E1 = 0.698). A considerable amount of E1 could still be detected on the surface of these cells as shown by immunofluorescence without permeabilization (Figure 5D(b)). 

A further representative construct, pYM-53, codes for a retention defective E1 protein, which contains six mutations (K174A, R177A, G178A, Q179A, Q182A, and G183A) in the TM region of E1 (TM sequence of the 6M mutant given in Figure 5A in [23]). The protein expressed from this construct was shown to be transported to the plasma membrane. When the E2 wt was co-expressed with this HA-tagged E1 mutant, E2 again followed the retention defective E1 mutant and was present on the plasma membrane (Figure 5E(a)) (Mcc E2 = 0.833, Mcc E1 = 0.787). Thus, this E1 mutant behaves like the chimera HA-tagged E1_ecto_ + VSV G _TMD_ (pYM-56) and again, heterodimer formation was still detectable for the 6M E1 mutant and E2 wt (Figure 5C, lane 7, E1 (6M) + E2 wt). 

The importance of heterodimer formation in this context was also demonstrated when the covalent disulfide linkage between E1 and the E2 mutant R355K was prevented by mutating Cys residues 123 and 171 in E1 (E1 mutant C123S/C171S) (Figure 5C, lane 6). In this setting, E1 lost the ability to compensate for the reduced E2 retention, leading to E2 R355K detection on the cell surface in addition to partial co-localization with the mutant E1 protein within the cell (Figure 5E(b)) (Mcc E2 = 0.474, Mcc E1 = 0.713). These data indicate that E1/E2 heterodimer formation is essential for the ability of E1 to compensate for the retention deficit of the E2 mutant.

Taken together, these results clearly demonstrate that E1 is dominant with regard to subcellular localization of the covalently linked E1/E2 heterodimer. 

### 2.6. Both C123 in E1 and C295 in E2 Are Important for Viral Infectivity

**Figure 6:**
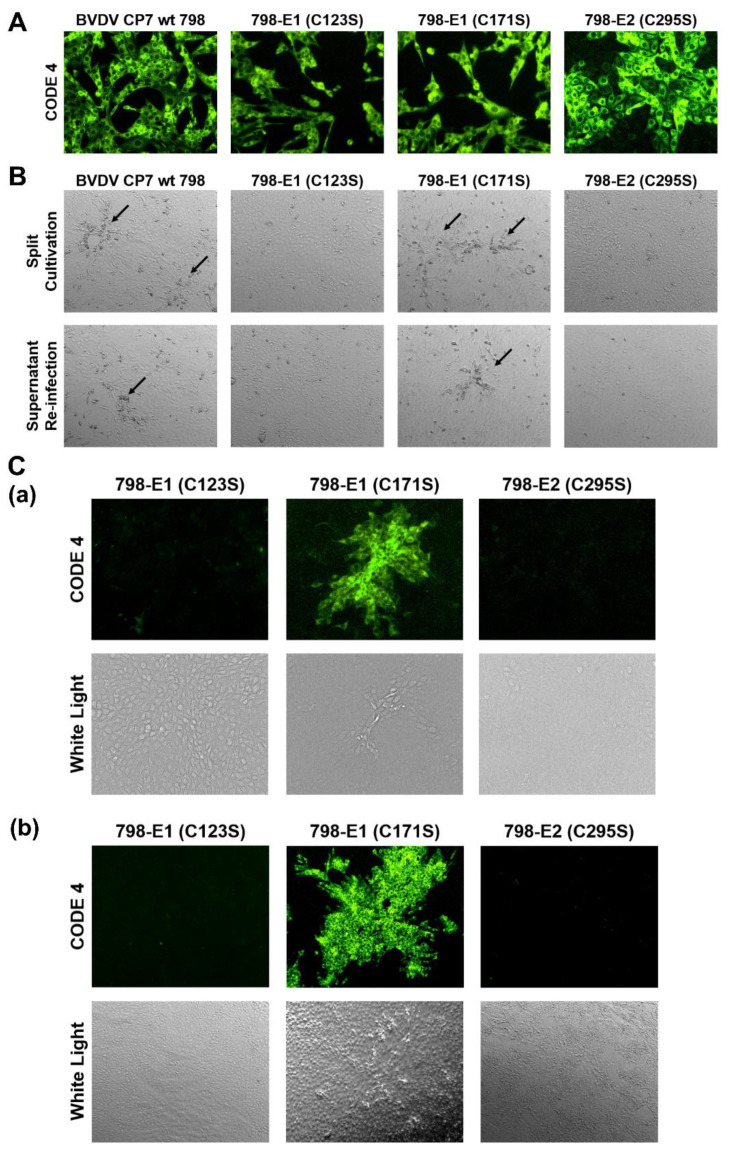
Effect of Cys mutations in E1 and E2 on recovery of infectious BVDV. (**A**) MDBK-B2 cells were first transfected by electroporation with the RNAs transcribed from the given plasmids. One day after EP, the cells were fixed with 4% PFA and permeabilized with 0.05% Triton X-100. The viral protein NS3 was detected with the primary antibody Code 4 and α-mouse FITC. (**B**) CPE observation after split of electroporated cells (upper row) or freeze/thaw extract infection of fresh cells (lower row). Arrows point at areas with rounded cells that show the beginning of a cytopathic effect (**C**) Analysis of cells split after electroporation (**a**) or of fresh cells infected with the supernatant (SN) of electroporated cells (**b**). The cells were fixed with 4% PFA and permeabilized with 0.05% Triton X-100. The viral protein NS3 was detected with mAb Code 4 (mAb 8.12.7 [37]) against NS3 and α-mouse FITC. Magnification 100×.

Mutation analysis allowed us to identify amino acids that have an influence on E1/E2 heterodimerization. In order to find out whether substitution of these residues interfered with the formation of infectious pestivirus particles, the mutations C171S and C123S in E1 as well as C295S in E2 were introduced into the BVDV CP7 full-length infectious clone p798 [34]. Using T7 RNA polymerase transcription, viral genome-like RNAs were synthesized and used for electroporation (EP) of MDBK-B2 cells. Cells were seeded in two cell culture dishes upon EP. Twenty-four hours after electroporation, one dish with electroporated cells of each sample was tested for viral protein expression using indirect immunofluorescence with the monoclonal antibody Code 4 directed against the pestivirus NS3 protein and anti-mouse FITC. For all samples, positive immunofluorescence signals were detected in the electroporated cells, proving that the in vitro transcribed viral genome-like RNAs were functional autonomous replicons, since in our system, translation of input RNA did not lead to detectable amounts of viral proteins (Figure 6A). The transfected cells were split and further incubated to be able to observe the typical CPE of BVDV CP7, detection of which would indicate cell to cell spread of virus. To check for the recovery of released infectious viruses, the supernatant (SN) of electroporated cells was collected and used for infection of fresh MDBK-B2 cells. As shown in Figure 6B, only transfection of RNA transcribed from p798 wt and p798-E1 C171S resulted in the detection of a typical CPE in both split and SN-infected cells. After RNA isolation and RT-PCR, the sequencing results confirmed that the recovered viruses were still carrying their corresponding substitution. No indication for reversion was found.

We conducted a further immunofluorescence analysis for all three transfections with mutated RNAs. This experiment showed that 798-E1 C123S and 798-E2 C295S were lost with the cell passage and could not be further enriched (Figure 6C). This finding strongly indicates that the transfected cells died as a consequence of the CPE induced by replicon replication as reported previously [35,36]. From this result, it can be concluded that these mutations interfered with generation of infectious viruses and time was too short for reversion to a viable variant. These findings further clarified that the above-mentioned two cysteine residues in E1 and E2 are critical for viral infectivity, which strongly suggests that the formation of covalently bound E1/E2 heterodimers is crucial for BVDV infectivity. It is worth noting that only a few fluorescent plaques could be observed two days after infection of fresh cells with SN from 798-E1 C171S electroporated cells whereas the SN from wt transfected cells yielded almost 100% positive cells. This indicates that the infectivity of BVDV CP7 containing the mutation C171S in E1 was also reduced. Nevertheless, the C171S mutation was stable in the recovered viruses for at least three passages. 

## 3. Discussion

Formation of oligomeric structures represents an important feature of many viral surface proteins and is often crucial for their functions, especially during infection. In pestiviruses, disulfide linked heterodimers composed of E1 and E2 are found in cells and virions [8], and mutations preventing heterodimer formation blocked the generation of infectious pseudotyped VSV [14]. E1 contains six cysteines in most pestiviral species and it is not known which of these residues are involved in intra- or intermolecular disulfide bond formation. The prediction from a computational model suggested that the last cysteine residue (Cys171) at the C-terminus of E1 could be engaged in intermolecular disulfide linkage [19]. However, the multiple sequence alignment throughout the pestiviral species A to K showed that C171 is not fully conserved. A C171F mutation is found in the species BVDV-2 and pronghorn antelope pestivirus, whereas porcine Bungowannah pestivirus displays a C171A exchange and pestivirus isolate J-NrPV/NYC-D23, a switch of the cysteine from position 171 to 170 with F at position 171 (see Supplementary Materials). Since the E1/E2 heterodimer is critical for pestiviral infectivity, as we show here, it is unlikely that C171 is the important cysteine residue for E1/E2 heterodimer formation. Additionally, C171 is located in the putative transmembrane domain and it is difficult to imagine how this residue could get in contact with the free thiol at position 295 of E2. In our study, we showed that C123 plays the essential role in both E1/E2 heterodimer formation and the oligomerization of E1. However, this finding is in contrast to that in another study, which suggested that both C24 and C94 of E1 are important for E1/E2 heterodimerization [20]. However, we cannot explain this discrepancy. Even though the respective study was done with CSFV and not BVDV, it is not likely that there is species dependent variation at this point. In our hands, mutation of those residues did not interfere with oligomer formation (Figure 2). Moreover, at least C24 is located in the N-terminal glycosylated part of E1 that can be supposed to be exposed and is therefore not prone to interaction with C295 of E2, which should be located close to the membrane [17,18]. 

One paper on the function of CSFV E1 demonstrated that individual Cys to Ser mutations had no effect on E1/E2 heterodimerization in infected cells [38], suggesting that more than one disulfide linkage could be contributing to the heterodimer formation of E1/E2. However, the intramolecular disulfide bonds in pestiviral E2 (BVDV as an example) have been clarified via crystal structure analysis [17,18]. As shown in Figure 2A, the E2 ectodomain contains 17 cysteine residues. Among them, the first 16 cysteine residues form eight intramolecular disulfide linkages. Accordingly, C295 is the only unpaired cysteine in E2, making it the logical candidate for both the E2 homodimer and E1/E2 heterodimer formation. Based on this consideration, it is unlikely that there is more than one defined intermolecular disulfide bond contributing to the heterodimer formation. In our study, we showed that C123 in E1 was the critical site for E1/E2 heterodimerization, whereas C171 was dispensable. The predicted distance between C123 in E1 and the border of the putative TM domain of E1 was similar to that of C295 in E2, so these two cysteine residues had a higher probability of getting in contact, whereas Cys171 was most likely hidden in the membrane. Moreover, the publication by Risatti and co-workers [38] also reported that the individual substitutions of Cys residues in CSFV E1 did not interfere with the infectivity of the virus in vivo, showing virulence features similar to those of the parental wt virus. In contrast, we showed here that the single mutation C123S in E1 triggered a defect of viral infectivity in tissue culture experiments. The detailed function of E1/E2 heterodimers in the viral life cycle is not known for either pesti- or hepaciviruses at the moment, but the mutation analyses carried out for both of these viruses strongly support the conclusion that a defect in dimer formation interferes with recovery of infectious viruses and thus points to a crucial role of these structures. 

Two envelope proteins of pestiviruses, namely E^rns^ and E2, have been shown to form covalently linked homodimers. However, there is a long-standing question of whether pestiviral E1 also forms homooligomers. In the present study, we showed for the first time the formation of dimers and trimers of E1. Even though the relative amounts of these forms varied between experiments for unclear reasons, it has to be noticed that we always found these forms. Since these structures were found only on SDS-PAGE under a non-reducing condition, the oligomers were obviously covalently linked via disulfide bonds. Our experiments, aimed at identifying the relevant cysteines, revealed that, again, C123 represents the most important residue for the homooligomerization of E1. However, in these experiments also replacement of C171 had an effect. Apparently, formation of both E1 homodimers and -trimers is somewhat reduced in a C171S mutant. The reduction of homodimer formation was stronger in a C123S mutant while E1 trimers could no longer be detected for this mutant. Importantly, the exchange of both C123 and C171 resulted in the complete absence of oligomeric forms. These results can be interpreted in a way that E1 homodimers are predominantly C123 linked whereas the trimer involves C171. Interestingly, we found no clear indication for higher oligomeric structures that could be obtained by connecting two or more C123 linked homodimers via their C171 residues, even though vague bands of higher molecular weight were detected in WBs, which disappeared upon reduction. We cannot explain the detection of E1 trimers or putative higher oligomers for mutant C171S or the truncated E1 proteins with less than 171 residues left. Thus, it has to be concluded that the basic structure of the E1 oligomers is somewhat obscure, even though the data demonstrate an important role of C123 and C171. 

In HCV, E1 forms a non-covalently linked SDS resistant trimer, which is thermally unstable. The conserved ‘GxxxG’ motif in the transmembrane region of HCV E1 has been shown to be critical for this trimerization [10]. Mutations affecting this motif hampered the generation of infectious HCV particles, indicating a crucial function of the E1 trimers. A GxxxG motif is missing in pestiviral E1, which relies on covalent disulfide linkage for E1 homooligomerization. It can, of course, not be excluded and it is even likely that in pestiviruses, non-covalent interaction also contributes to the establishment of oligomeric structures of the envelope proteins. However, in contrast to HCV, such interactions would have to be much weaker so that their detection on denaturing gels is not possible. Thus, the molecular basis of E1 homooligomerization is clearly different between pestiviruses and HCV. This is also true for E1/E2 heterodimerization, which in HCV infected cells is again non-covalent, but disulfide-based in pestiviruses. It has, however, to be stated that larger covalently linked oligomeric forms of HCV glycoproteins have been demonstrated in infectious virus particles, so the covalent linkage of envelope proteins also seems to be important in HCV [39]. Another striking difference between HCV and pestivirus E1 oligomerization is the dependence of the former on the presence of E2 or the E2 TM domain [10]. 

Interestingly, we found that E1 can overrule the retention signal of E2 in E1/E2 heterodimers. In other words, the subcellular localization of E2 largely depends on that of E1 when the covalent linkage exists. In contrast, in the heterodimer, E2 was not able to overrule E1 with regard to its cellular localization. A single mutation at R355 or Q370 in E2 can suppress its retention [21,22], making E2 retention vulnerable to mutations. It is worth noting that even though R355K in E2 can result in a partial retention defect, the natural pestivirus isolate BVDV NewYork’93 [40] contains this substitution, indicating that this mutation at position 355 is tolerable for the virus. In contrast, the retention of E1 relies on a hexamer of polar amino acids in the center of the E1 TMD, and a variety of mutants with changes of this sequence were still retained within the cell as long as one or two polar residues were preserved [23]. Thus, the retention system of E1 is obviously very stable and not prone to mutation induced destruction since different shorter parts of it are sufficient for intracellular retention. One could consider that pestiviruses use this complex and stable retention signal as a ‘safety’ mechanism, ensuring that E2 via its linkage to E1 can still be processed in the ER as usual, even when some unpredictable mutations destroy its own retention signal. A similar phenomenon was reported for the complex between pre-membrane (prME) and envelope (E) of YFV (yellow fever virus), in which the prME heterodimer contained several retention signals of different strength [41]. In pestiviruses, the conserved six polar residues in the E1 TMD affecting its intracellular localization certainly have additional functions beyond retention since mutations of part of these residues prevented the recovery of infectious viruses even though E1 was retained in the ER [23].

In our study, the E1/E2 heterodimer was always detected with specific antibodies directed against E2. We tried several antibodies against the HA-tag introduced at the amino-terminus of E1. Unfortunately, none of them could be used for the detection of the E1/E2 heterodimer even though these reagents worked very well when E1 was expressed alone. It seems that when E1 is co-expressed with E2, the N-terminal HA-tags were somehow shielded by E2 after the folding of both proteins, so that the HA epitope was not detectable. This phenomenon raises questions about the 3D structure of the heterodimer, which should be determined not only with regard to this point, but also to understand pestivirus infection. 

The primary interaction of the two partners in hetero- or homodimers of viral envelope proteins is often established by the TM regions [27,42,43]. It has been shown that substitutions in positions K174 and R177 in E1 play a role in pestivirus E1/E2 heterodimer formation [14]. However, we observed that the K174A/R177A double mutation in E1 had no significant effect on the E1/E2 heterodimerization. Even the protein expressed from plasmid pYM-53, in which all polar residues in the TM region of E1 were removed, could still form E1/E2 heterodimers when co-expressed with E2 wt (Figure 5C, lane 7). Surprisingly, we even found that the formation of the E1/E2 heterodimer did not absolutely require the presence of the E1 TMD, so that lower amounts of heterodimer were still generated when wt E2 was co-expressed with the E1 ectodomain alone. Similarly, E1 still formed E1 oligomers in the absence of its TMD, showing that the TM sequence is not the main driver for interaction of the E1 monomer with other envelope protein molecules. Since the TMD contains C171, these results further prove that this residue is dispensable for the interaction between E1 and E2. 

Taken together, our data revealed interesting similarities, but also differences between E1 of pestiviruses and E1 of HCV with regard to homo- and heterooligomerization and its interplay with E2. Further molecular studies are needed to fully understand the structure and function of E1 containing oligomers. Only with additional research on E1 will the mechanisms involved in pestiviral membrane fusion, assembly, and budding be elucidated. 

## 4. Materials and Methods

### 4.1. Cells and Antibodies

Baby hamster kidney (BHK)-21 cells (kindly provided by T. Rümenapf, University of Veterinary Medicine, Vienna, Austria), rabbit kidney epithelial cells (RK)13 cells (ATCC#: CCL-37), and clone B2 established from Madine-Darby bovine kidney (MDBK)-cells (ATCC#: CCL-22) were grown in Dulbecco’s modified Eagle’s medium supplemented with 10% fetal calf serum and nonessential amino acids at 37 °C in 5% CO_2_. For cultivation of MDBK-B2 cells, the fetal calf serum (FCS) was specifically BVDV free (tested for the absence of viruses and antibodies against BVDV). For the confocal immunofluorescence, cells were seeded on coverslips one day before transfection.

Primary antibodies used for immunofluorescence were α-HA tag antibody ([HA.C5] ab18181, Abcam, Cambridge, UK), α-BVDV E2 BVDV-Mix (four mouse monoclonal antibodies 1a16, 1b8, 1b31, D5 [33]), panpesti mAb Code 4 (mAB 8.12.7. [37]) directed against NS3, and mAb f48, established against CSFV E2, but cross-reacting with E2 from BVDV CP7 [44]. Secondary antibodies used for immunofluorescence were α-mouse-Alexa Fluor 488, α-rabbit-Alexa Fluor 594, α-mouse-IgG2a-Alexa Fluor 488, α-mouse-IgG2b-Alexa Fluor 488, α-mouse-IgG2a-Alexa Fluor 594 (all Lifetechnology, Munich, Germany), α-mouse-IgG1-Cy3, and α-mouse-FITC (both Dianova, Hamburg, Germany). Antibodies used for western blotting were WB214 (Veterinary Laboratories Agency, Weybridge, UK), α-HA (ab137838 anti-rabbit serum, Abcam Cambridge, UK), α-mouse-HRPO, and α-rabbit-HRPO (Dianova, Hamburg, Germany). 

### 4.2. Cloning

pCR-13 and pCR-16 have been described before [22]. Both constructs are based on plasmid pCI (Promega, Walldorf, Germany). pCR-13 contains the cDNA coding for BVDV CP7 E1 with E^rns^ signal sequence. pCR-16 contains the cDNA coding for E2 with its Amino-terminal signal sequence and a Carboxy-terminal AU1-tag. pYM-13 is based on pCR-13 with the sequence coding for the HA-tag (YPYDVPDYA) inserted at the 5′end of the E1 gene downstream of the signal sequence coding sequence. Point mutations were introduced by Quick change PCR-based mutagenesis, as recommended by Promega (Walldorf, Germany). pCR-78 and pCR-79 are based on pCR-16, with mutations changing an arginine to a lysine codon or with a deletion of the arginine codon at position 355 in the BVDV CP7 E2 coding sequence. pYM-56, pYM-71, pYM-72, and pYM-75 are all based on pYM-13 (HA-tagged E1), in which the TM domain of E1 was fully or partially replaced by that of VSV G, leading to the expression of chimeric proteins with the HA tagged CP7 E1 ectodomain on the cell surface [23]. Selected mutated sequences were transferred from E1 or E2 expression plasmids to pA/BVDV, the infectious clone for BVDV CP7 [34], using restriction enzymes according to standard procedures [45]. All plasmids were verified by nucleotide sequencing. Further details of the cloning procedures including primer and plasmid sequences are available on request.

### 4.3. Transient Expression and Analysis of Proteins

Twenty-four hours before transfection, BHK-21 or RK-13 cells were seeded in 24-well or 6-well plates at about 60% confluency. BHK-21 and RK-13 cells were transfected with expression plasmids using Lipofectamine 2000 (Invitrogen) according to the manufacturer’s recommendations. 

For detection of the expressed proteins via western blot, the cells were lysed 24 h post transfection with the addition of 50 μL of reducing 1x SDS sample buffer (120 mM Tris-HCl, pH 6.8, 20% glycerol, 4% SDS, 0.02% Bromphenol Blue) containing 5% β-mercaptoethanol. The proteins were separated by SDS-PAGE [46] and subsequently transferred from the gel to a nitrocellulose membrane (GE Healthcare/Fisher Scientific, Schwerte, Germany) with the Bio-Rad Mini Trans-Blot^®®^ system (Bio-Rad, München, Germany). A nitrocellulose (NC) membrane with a pore size of 0.2 μm was used. Transfer was done at 100 V for 1 h. To detect the target proteins on a nitrocellulose membrane, it was first incubated for one hour with block buffer [5% (*w*/*v*) Low-fat milk powder (BD Biosciences, Heidelberg, Germany) in PBS-T] to saturate non-specific binding sites. After blocking the membrane and a short washing step with PBS-T [0.5% (*v*/*v*) Tween-20 (Sigma-Aldrich, Hamburg, Germany)], the detection antibody was diluted with PBS-T solution, incubated overnight at 4 °C on the shaker. The next day, the NC membrane was washed three times for 10 min with PBS-T and then incubated for 1–2 h with a suitable secondary antibody at room temperature. After washing with PBS-T (three times, 10 min), the proteins were detected using the SuperSignalTM West Pico Chemiluminescent Substrate Kit (Thermo Fisher Scientific Inc., München, Germany) as recommended by the supplier. Briefly, 5× volume PBS-T plus two components of the kit were mixed 1:1 and then the membrane was incubated with the solution for 2 min in the dark. Then, a ChemiDoc imaging system (ChemiDoc XRS+, Bio-Rad, München, Germany) was used for the detection of luminescence and Image Lab software (Version 6.1, Bio-Rad) for further analysis.

For immunoprecipitation, the proteins were expressed from transfected plasmids using the Vaccinia virus MVA-T7 expression system [30,31]. Briefly, BHK-21 cells were pre-infected with vaccinia MVA-T7 at a MOI of 5, transfected with expression plasmids coding for E1/E1 mutants or E2 or with pCITE empty vector by using SuperFect (Qiagen, Hilden, Germany) 1 h later. The expressed proteins were labelled with Tran35S-Label (Hartmann Analytics, Göttingen, Germany) as described [11]. Supernatant of the cell cultures (SN) was collected for the determination of secreted proteins, the cells were washed three times with PBS before lysis in RIP buffer. Both cell lysate (CL) and supernatant samples were prepared under denaturing conditions as described [11]. The produced proteins in SN/CL reacted with a specific anti-serum directed against the HA-tag or AU1-tag were precipitated. The samples were separated using SDS-PAGE under reducing conditions [46] and the labelled proteins were detected on imaging plates using a CR-35 Bio image plate scanner and AIDA Image Analyser 5.0 software (equipment and software from Elysia-Raytest, Straubenhardt, Germany).

For immunofluorescence, cells were fixed 24 h after transfection or infection with 4% PFA in PBS for 30 min either left untreated (non-permeabilized) or permeabilized in 0.05% Triton X-100 in PBS for 15 min at 4 °C. Both primary and secondary antibodies were diluted in PBS 10% FCS (E1: α-HA tag); isotype specific secondary antibodies were used in double labelling strategies using different mouse mAb; and coverslips were mounted in Mowiol medium with DAPI, and cells visualized on a Leica SP5 confocal laser scan microscope (×63 objective; numerical aperture 1.4, pinhole: 1 airy unit) with sequential acquisition on the fluorophores in multi-labelled samples. After acquisition colocalization was quantified by calculating the Mander’s coefficient (Mcc) for signal overlap using the JACoP_2.0 plugin [47] and thresholds for the images. Mccs were calculated from several images and means of those are given in this paper (not the Mcc calculated for the representative image shown). Mccs show the overlap of E1 signal with E2 signal (Mcc E1) or the overlap of the E2 signal with the E1 signal (Mcc E2) and were chosen because they better reflect the redistribution of one of the proteins than the Pearson’s coefficient for the same image. 

### 4.4. Virus Rescue

Virus rescue was essentially done as described previously [34]. Briefly, the full length plasmids based on pA/BVDV were linearized using SmaI. RNA was generated from these templates by in vitro transcription using a RiboMax-T7 large scale RNA production system (Promega). MDBK-B2 cells were freshly seeded into a 10 cm diameter dish one day before transfection and left to grow overnight to 100% confluence. On the day of electroporation, cells were harvested using trypsin, washed once with PBS, counted, and 10^5^ cells were resuspended in cold PBS for the electroporation with Hoefer Progenitor (Hoefer Inc. Holliston, MA, USA). The cell suspension was mixed with the appropriate RNA and electroporated with a single 1700 V pulse. Cells were resuspended in 5 mL medium, 50 µL transferred to a 96-well plate for immunofluorescence staining with monoclonal antibody 8.12.7 [37,48], kindly provided by E. J. Dubovi (Cornell University, Ithaca, NY, USA) after 48 h, and the remaining cells seeded in 24-well plates for cultivation. Infectious virus was harvested after three freeze–thaw cycles.

### 4.5. RNA Isolation and RT-PCR

Total RNA was extracted from infected cells in order to be able to analyze its sequence. For this purpose, MDBK B2 cells were infected 2–4 days before RNA extraction. The RNA was extracted with Trizol^®^ Reagent according to the manufacturer’s protocol (Invitrogen, Karlsruhe, Germany), and then stored at −20 °C until used for RT-PCR with the SuperScript™ III One-Step RT-PCR System Kit (Thermo-Fisher) as per the manufacturer’s protocol for the RT-PCR. The PCR products obtained in this way were first checked using agarose gel electrophoresis, then purified by preparative agarose gel electrophoresis and finally used for sequencing using the BigDye Terminator Cycle Sequencing Kit (PE Applied Biosystems, Weiterstadt, Germany). Sequence analysis and alignments were done with Geneious PrimeR software (Geneious Prime 2019.2.3) (Geneious Biomatters, Aukland, New Zealand).

Primers used in this section for RT-PCR: Seq BVDV E1 5′F: 5′-GTCACTTGTCGGAGGTGCTACTACTC-3′; Seq BVDV E1 3′R: 5′-GGGGTACCCTTGCGCCCCTGTTATG-3′ were purchased from Metabion (München, Germany).

## Data Availability

All data are provided in the manuscript.

## Supplementary Materials

The following are available online at https://www.mdpi.com/article/10.3390/ijms22147285/s1.
