# Supplementary material for: Interaction of Pestiviral E1 and E2 Sequences in Dimer Formation and Intracellular Retention
Source: Int J Mol Sci. 2021 Jul 6;22(14):7285. doi: 10.3390/ijms22147285 (PMC8306095; doi:10.3390/ijms22147285)
Supplement: Supplementary file 1 [file ijms-22-07285-s001.zip › ijms-1266080-suppl/ijms-1266080-suppl-resubmitetd.pptx]

## Slide 1
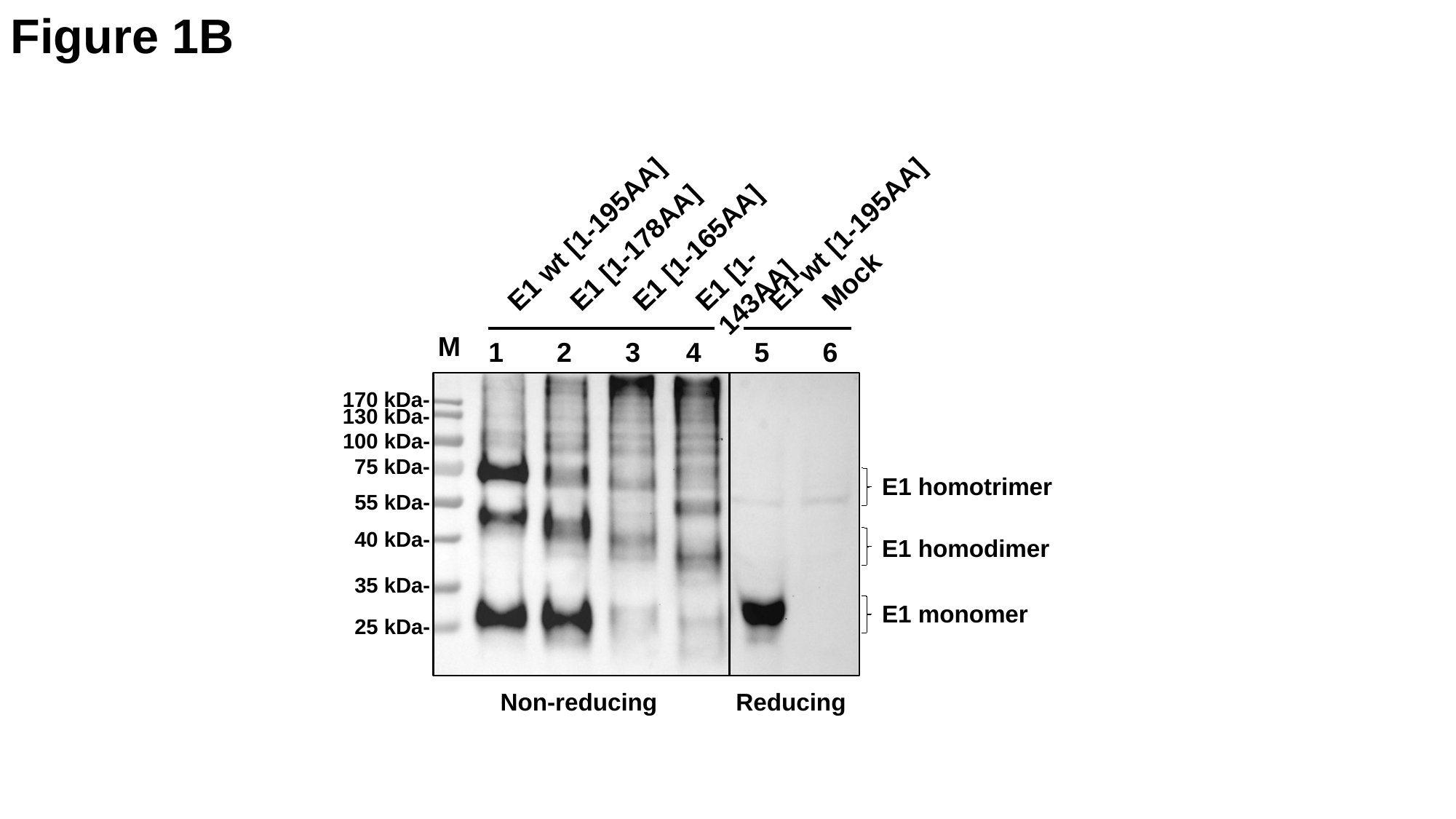

Figure 1B
E1 wt [1-195AA]
E1 wt [1-195AA]
E1 [1-178AA]
E1 [1-165AA]
Mock
E1 [1-143AA]
M
1 2 3 4 5 6
170 kDa-
130 kDa-
100 kDa-
75 kDa-
55 kDa-
40 kDa-
35 kDa-
25 kDa-
E1 homotrimer
E1 homodimer
E1 monomer
Non-reducing
Reducing

## Slide 2
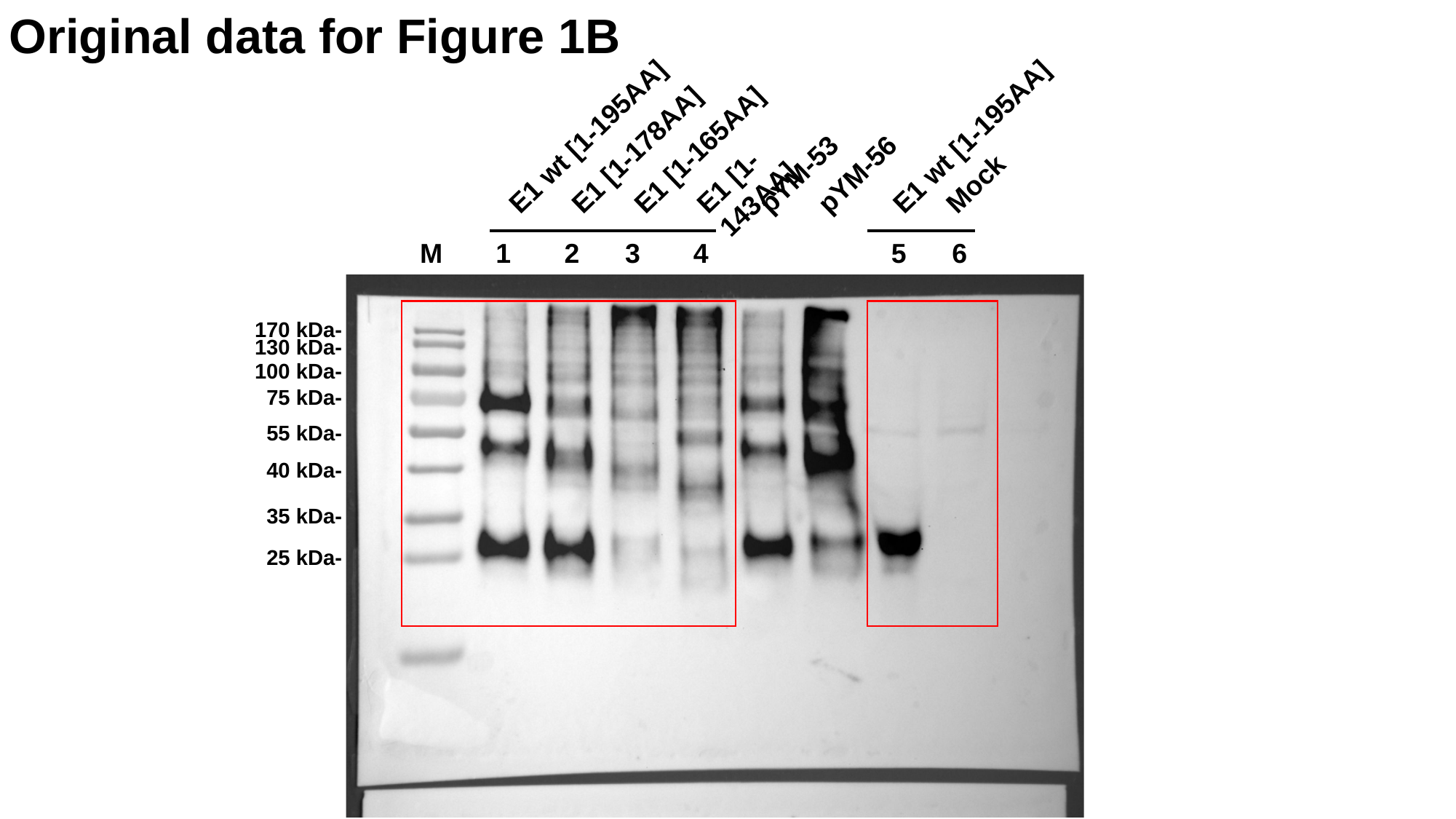

Original data for Figure 1B
E1 wt [1-195AA]
E1 [1-178AA]
E1 [1-165AA]
E1 [1-143AA]
pYM-53
pYM-56
E1 wt [1-195AA]
Mock
 M 1 2 3 4 5 6
170 kDa-
130 kDa-
100 kDa-
75 kDa-
55 kDa-
40 kDa-
35 kDa-
25 kDa-

## Slide 3
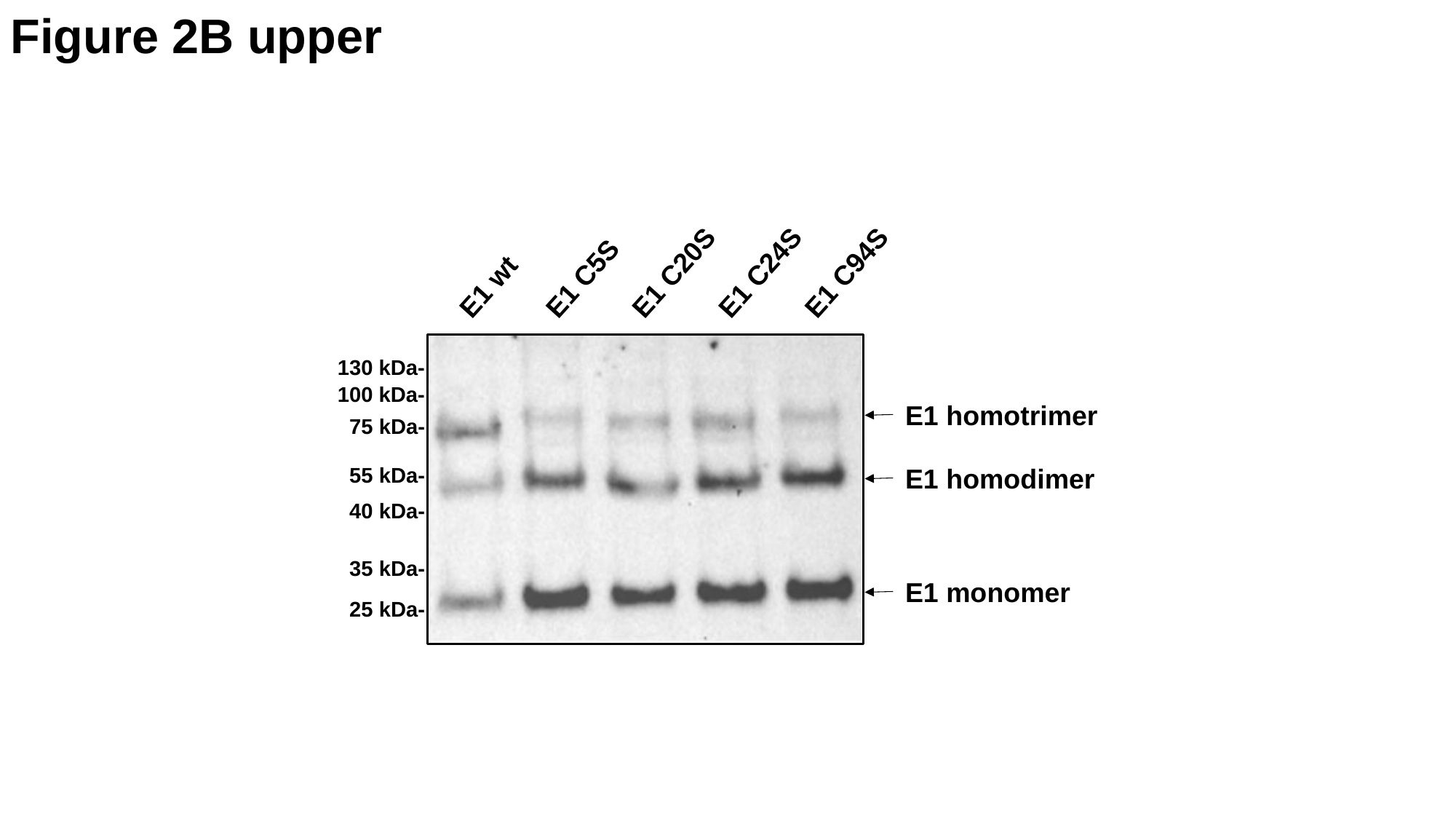

Figure 2B upper
E1 wt
E1 C5S
E1 C20S
E1 C24S
E1 C94S
130 kDa-
100 kDa-
75 kDa-
55 kDa-
40 kDa-
35 kDa-
25 kDa-
E1 homotrimer
E1 homodimer
E1 monomer

## Slide 4
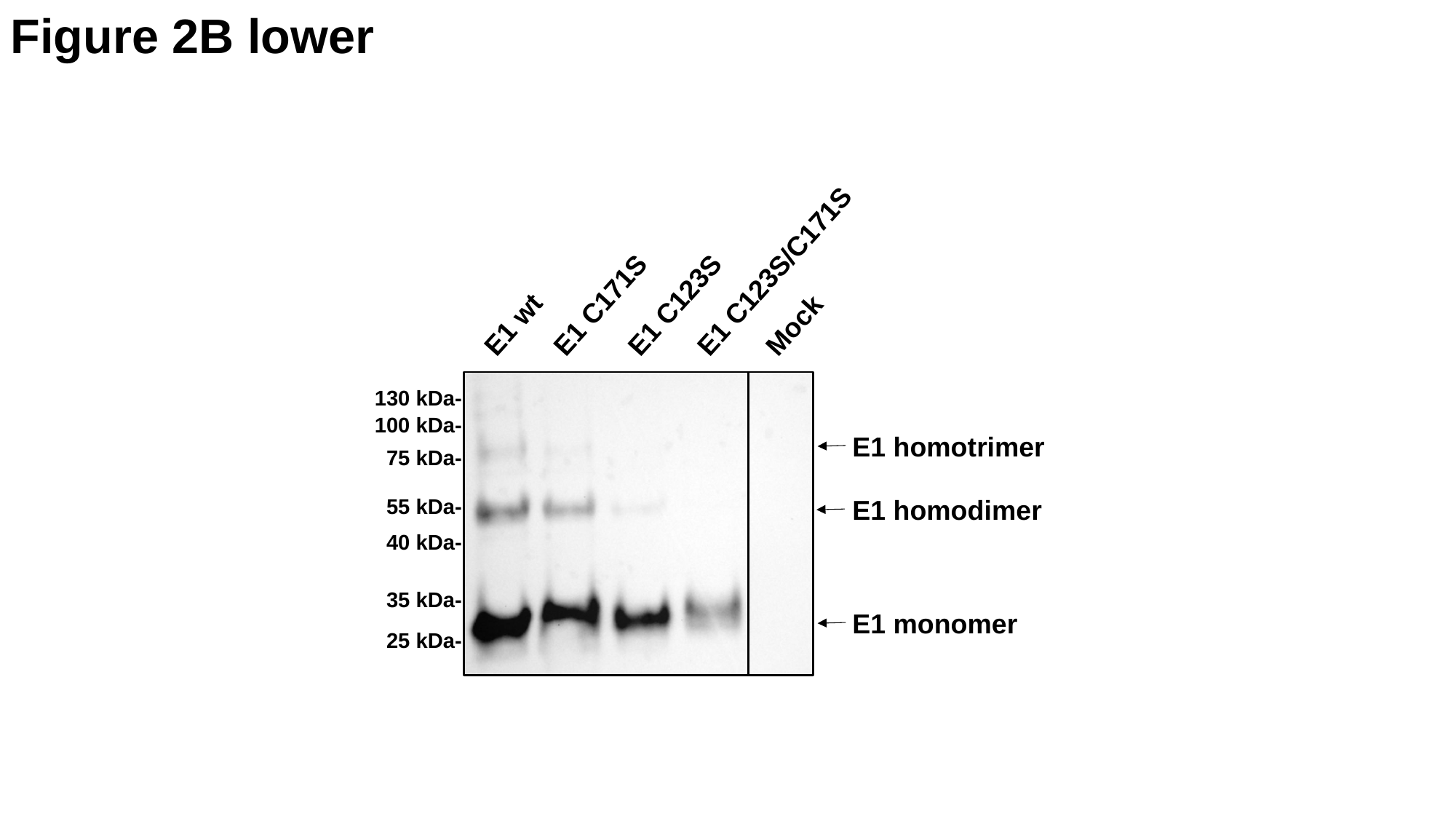

Figure 2B lower
E1 C123S/C171S
E1 wt
E1 C171S
E1 C123S
Mock
130 kDa-
100 kDa-
75 kDa-
55 kDa-
40 kDa-
35 kDa-
25 kDa-
E1 homotrimer
E1 homodimer
E1 monomer

## Slide 5
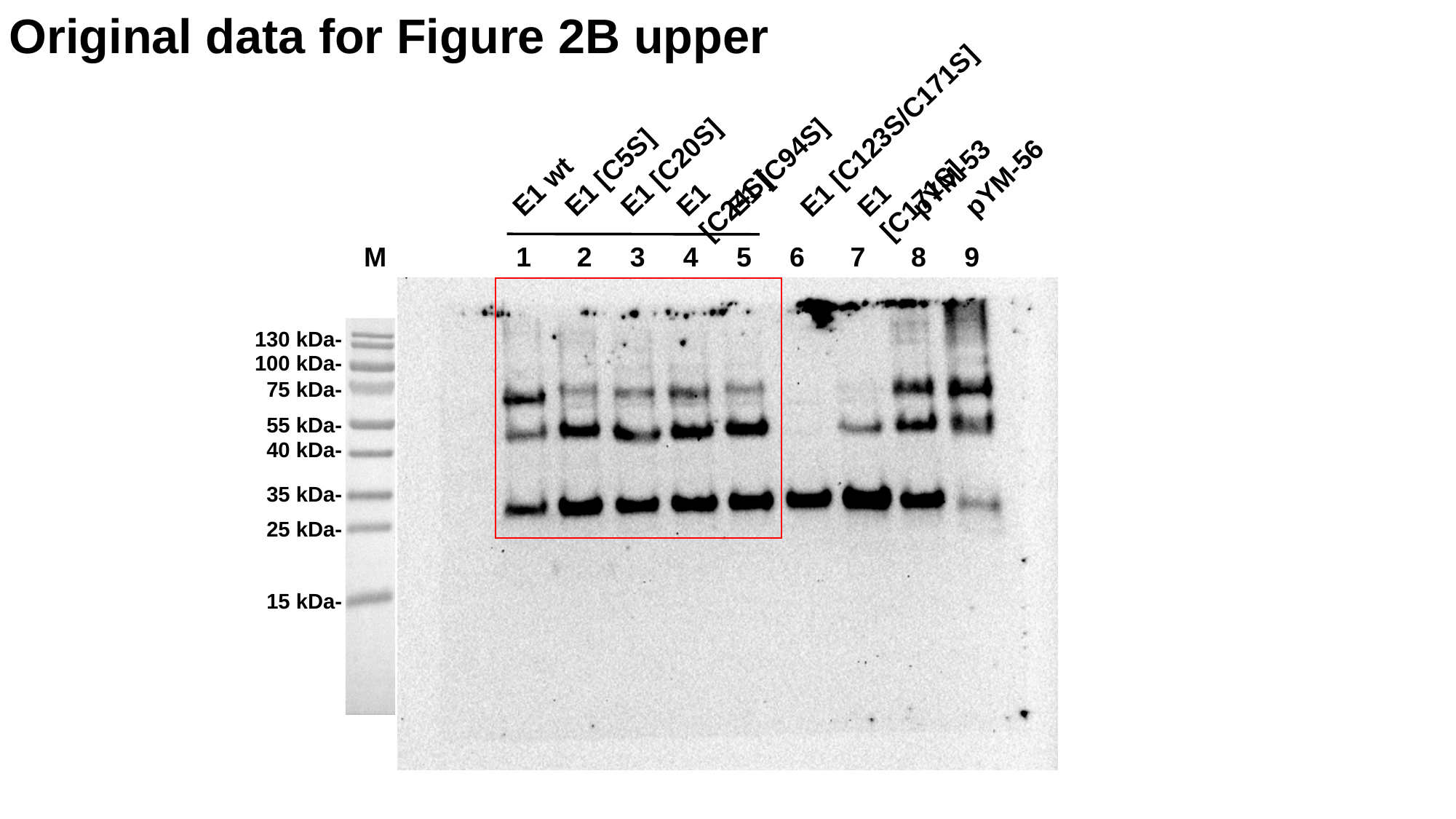

Original data for Figure 2B upper
E1 [C123S/C171S]
E1 [C5S]
E1 [C20S]
E1 [C94S]
E1 [C171S]
E1 [C24S]
pYM-53
pYM-56
E1 wt
 M 1 2 3 4 5 6 7 8 9
130 kDa-
100 kDa-
75 kDa-
55 kDa-
40 kDa-
35 kDa-
25 kDa-
15 kDa-

## Slide 6
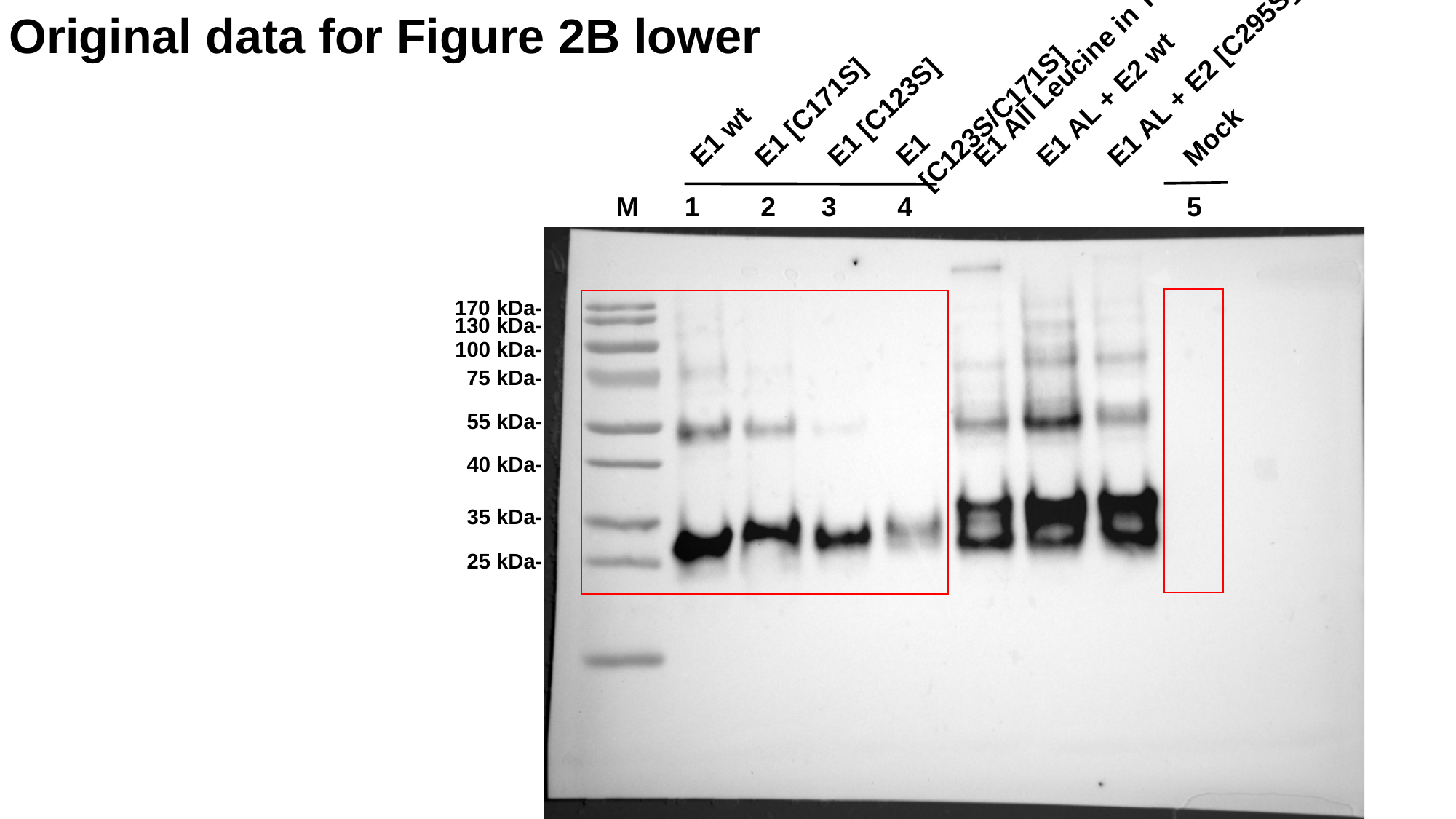

Original data for Figure 2B lower
E1 All Leucine in TM
E1 AL + E2 [C295S]
E1 [C123S/C171S]
E1 AL + E2 wt
E1 [C171S]
E1 [C123S]
Mock
E1 wt
 M 1 2 3 4 5
170 kDa-
130 kDa-
100 kDa-
75 kDa-
55 kDa-
40 kDa-
35 kDa-
25 kDa-

## Slide 7
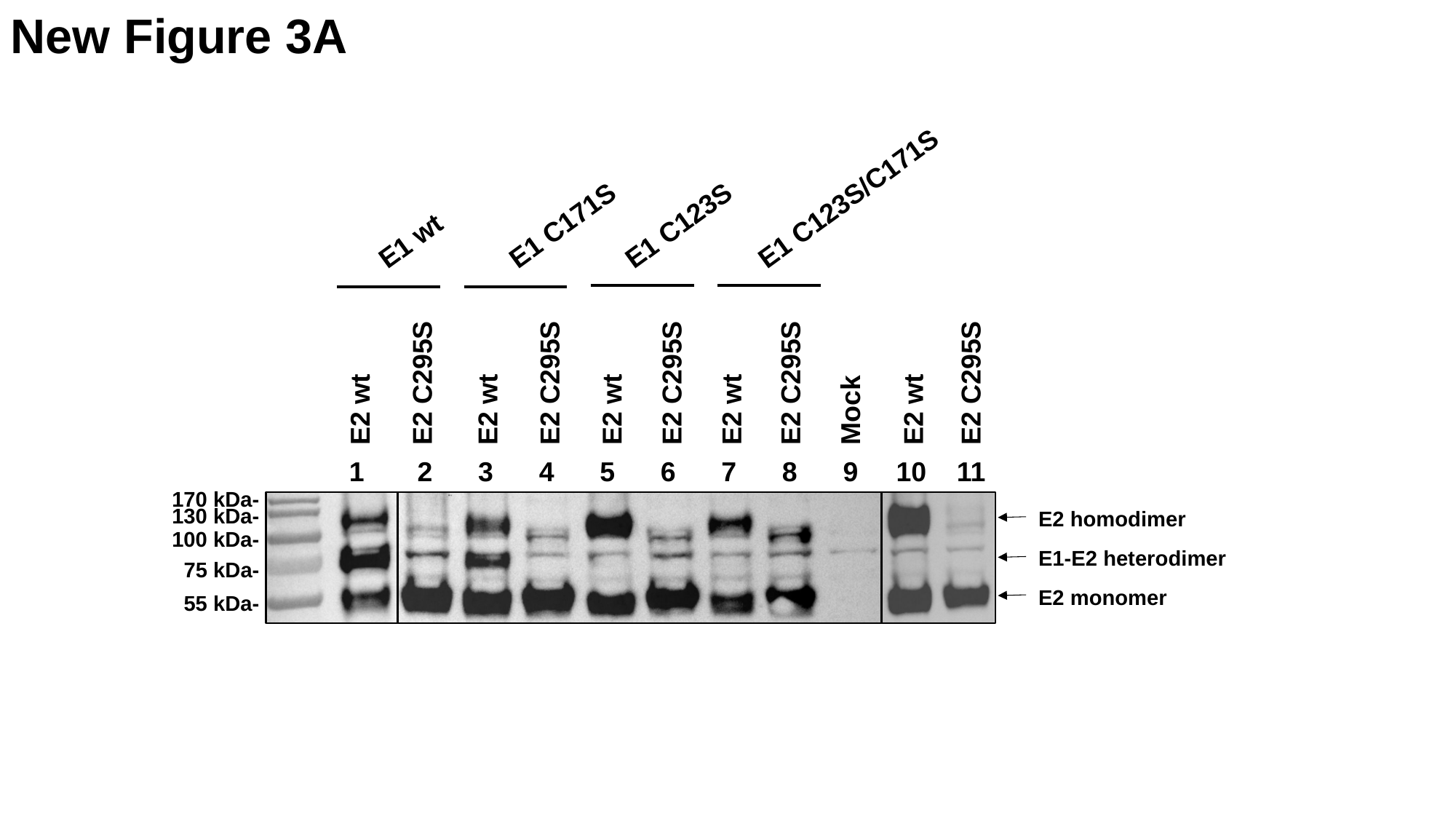

New Figure 3A
E1 C123S/C171S
E1 C171S
E1 C123S
E1 wt
E2 C295S
E2 wt
E2 C295S
E2 wt
E2 C295S
E2 wt
E2 C295S
E2 wt
E2 C295S
E2 wt
Mock
1 2 3 4 5 6 7 8 9 10 11
170 kDa-
130 kDa-
100 kDa-
75 kDa-
55 kDa-
E2 homodimer
E1-E2 heterodimer
E2 monomer

## Slide 8
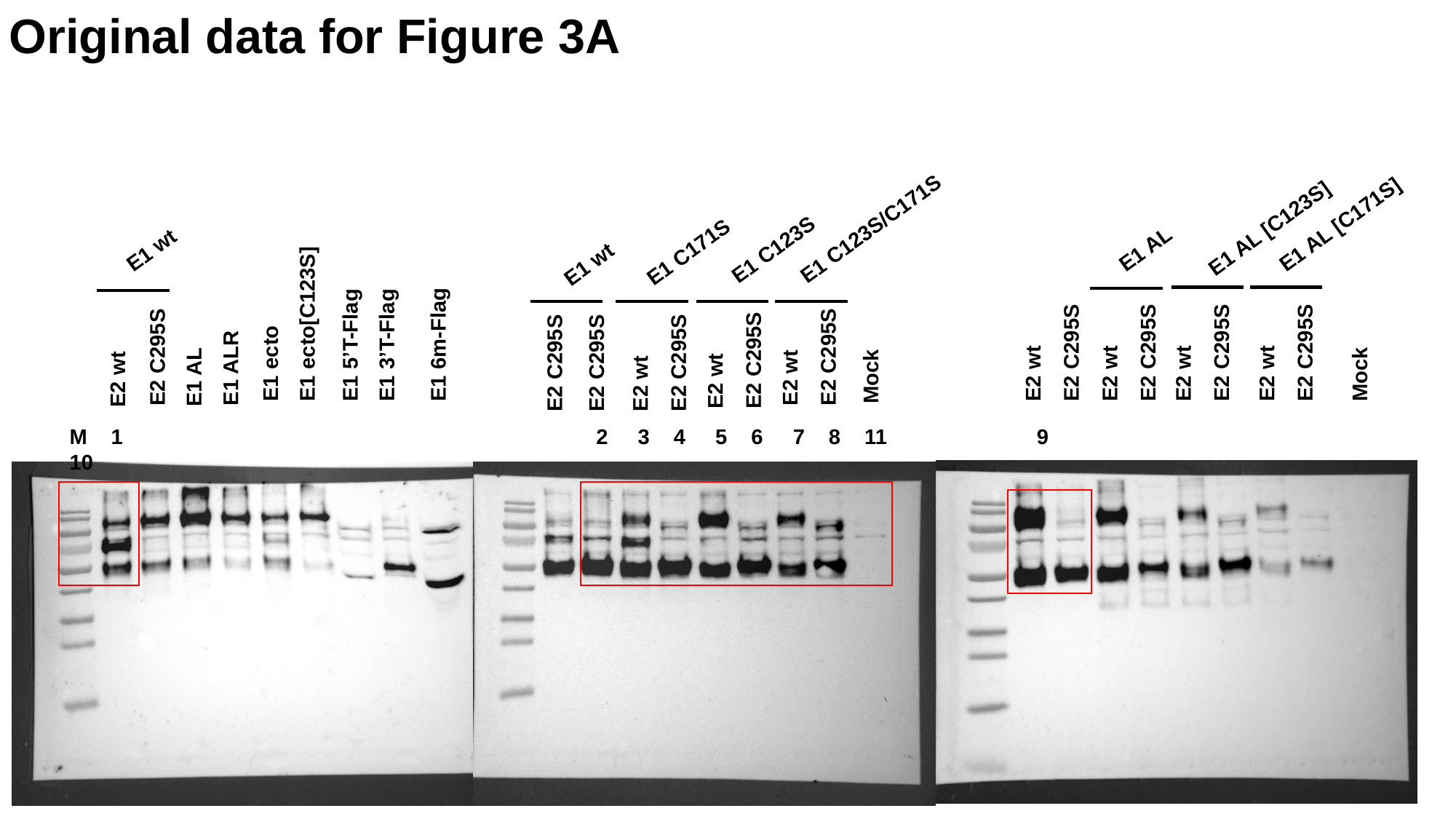

Original data for Figure 3A
E1 C123S/C171S
E1 AL [C171S]
E1 AL [C123S]
E1 AL
E1 wt
E1 C123S
E1 C171S
E1 wt
E2 C295S
E2 wt
E2 C295S
E2 wt
E2 C295S
E2 wt
E2 C295S
E2 wt
E2 C295S
E2 wt
E2 C295S
E2 wt
E1 ecto[C123S]
E1 5’T-Flag
E1 3’T-Flag
E1 6m-Flag
E2 C295S
E2 wt
E1 ecto
Mock
Mock
E1 ALR
E1 AL
E2 wt
E2 C295S
E2 C295S
E2 C295S
M 1 2 3 4 5 6 7 8 11 9 10

## Slide 9
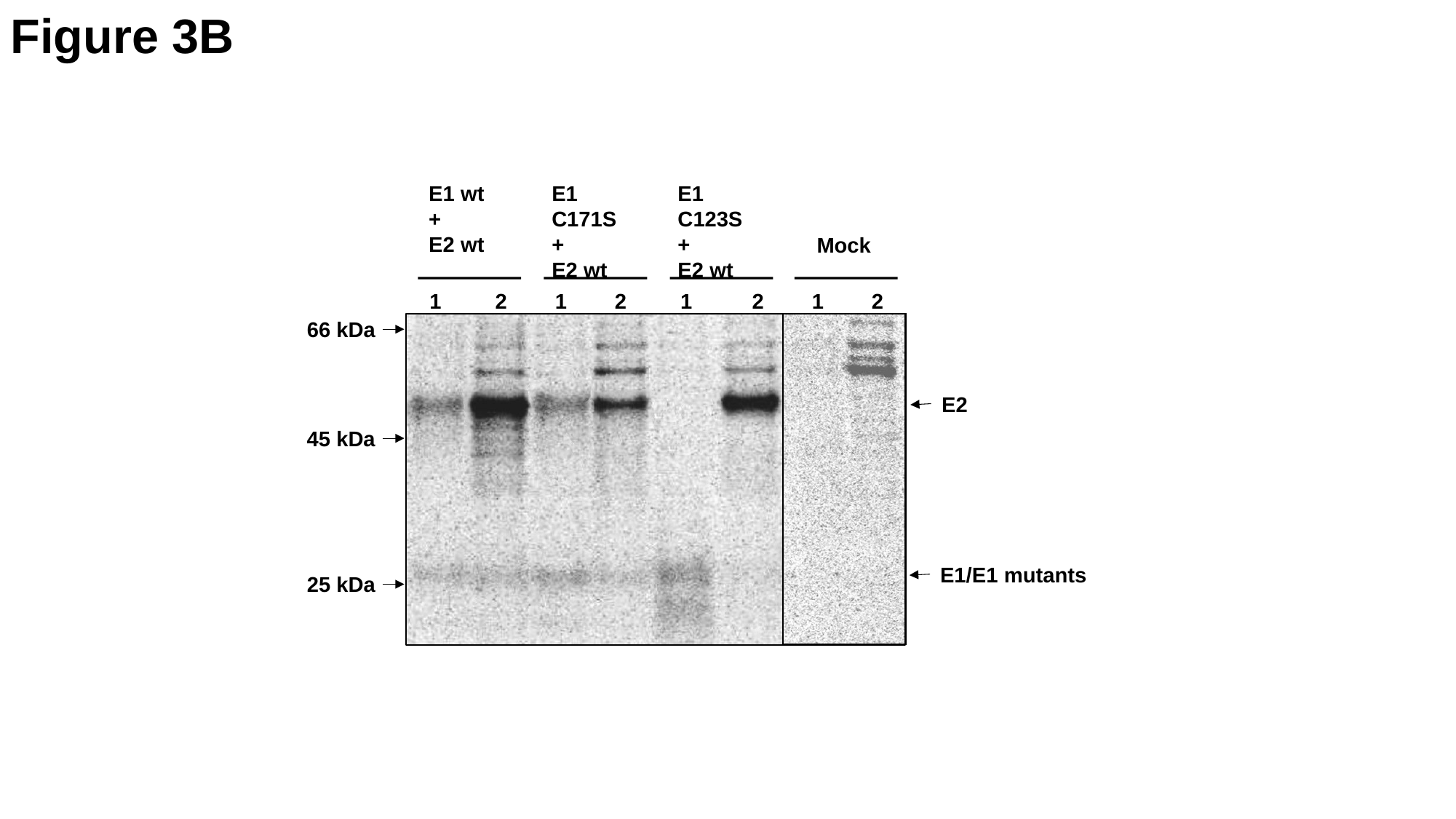

Figure 3B
E1 wt
+
E2 wt
E1 C171S
+
E2 wt
E1 C123S
+
E2 wt
Mock
 1 2 1 2 1 2 1 2
66 kDa
E2
45 kDa
E1/E1 mutants
25 kDa

## Slide 10
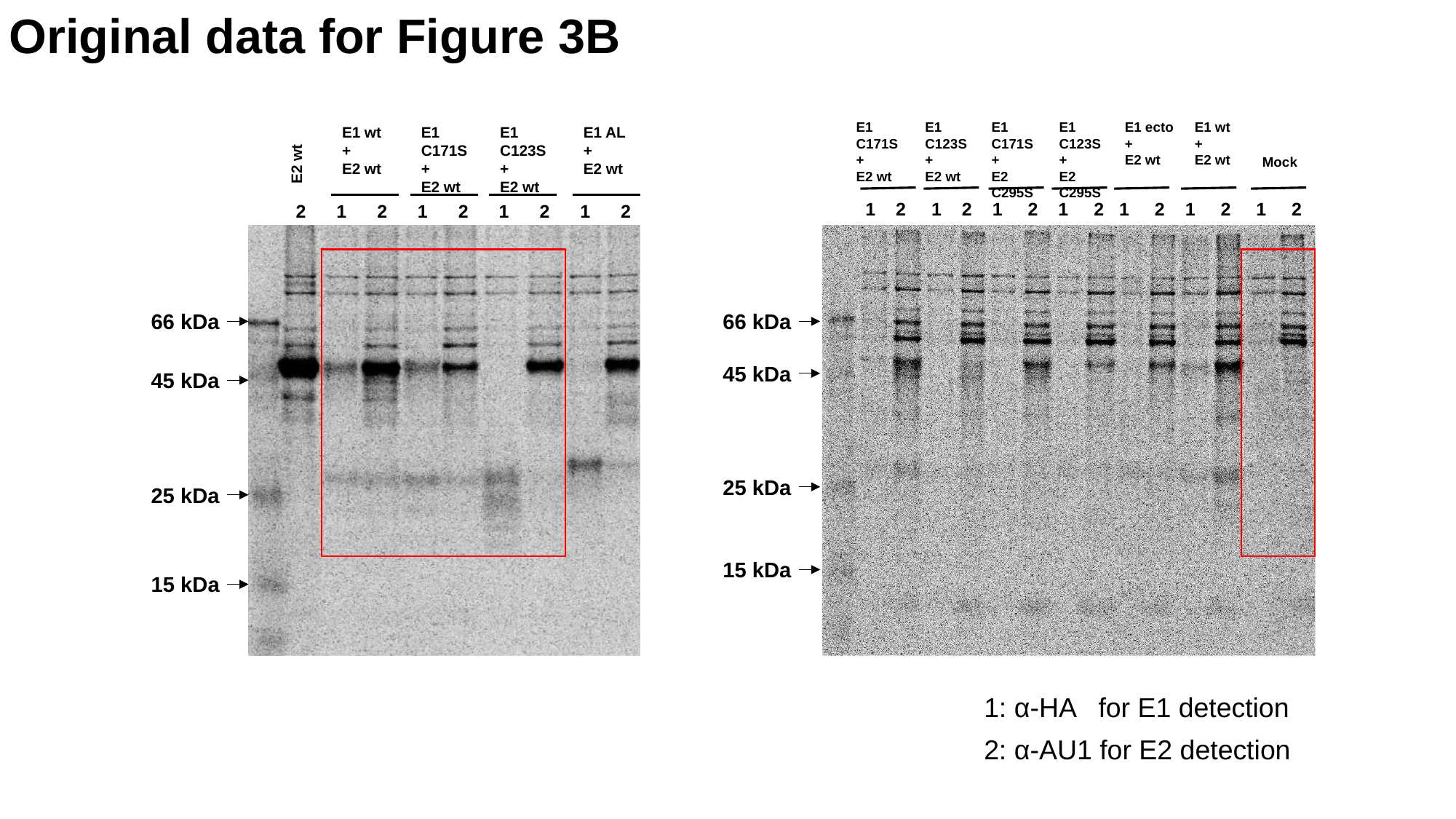

Original data for Figure 3B
E1 C123S
+
E2 wt
E1 C171S
+
E2 wt
E1 C171S
+
E2 C295S
E1 C123S
+
E2 C295S
E1 ecto
+
E2 wt
E1 C123S
+
E2 wt
E1 AL
+
E2 wt
E1 C171S
+
E2 wt
E1 wt
+
E2 wt
E1 wt
+
E2 wt
Mock
E2 wt
 1 2 1 2 1 2 1 2 1 2 1 2 1 2
 2 1 2 1 2 1 2 1 2
66 kDa
66 kDa
45 kDa
45 kDa
25 kDa
25 kDa
15 kDa
15 kDa
1: α-HA for E1 detection
2: α-AU1 for E2 detection

## Slide 11
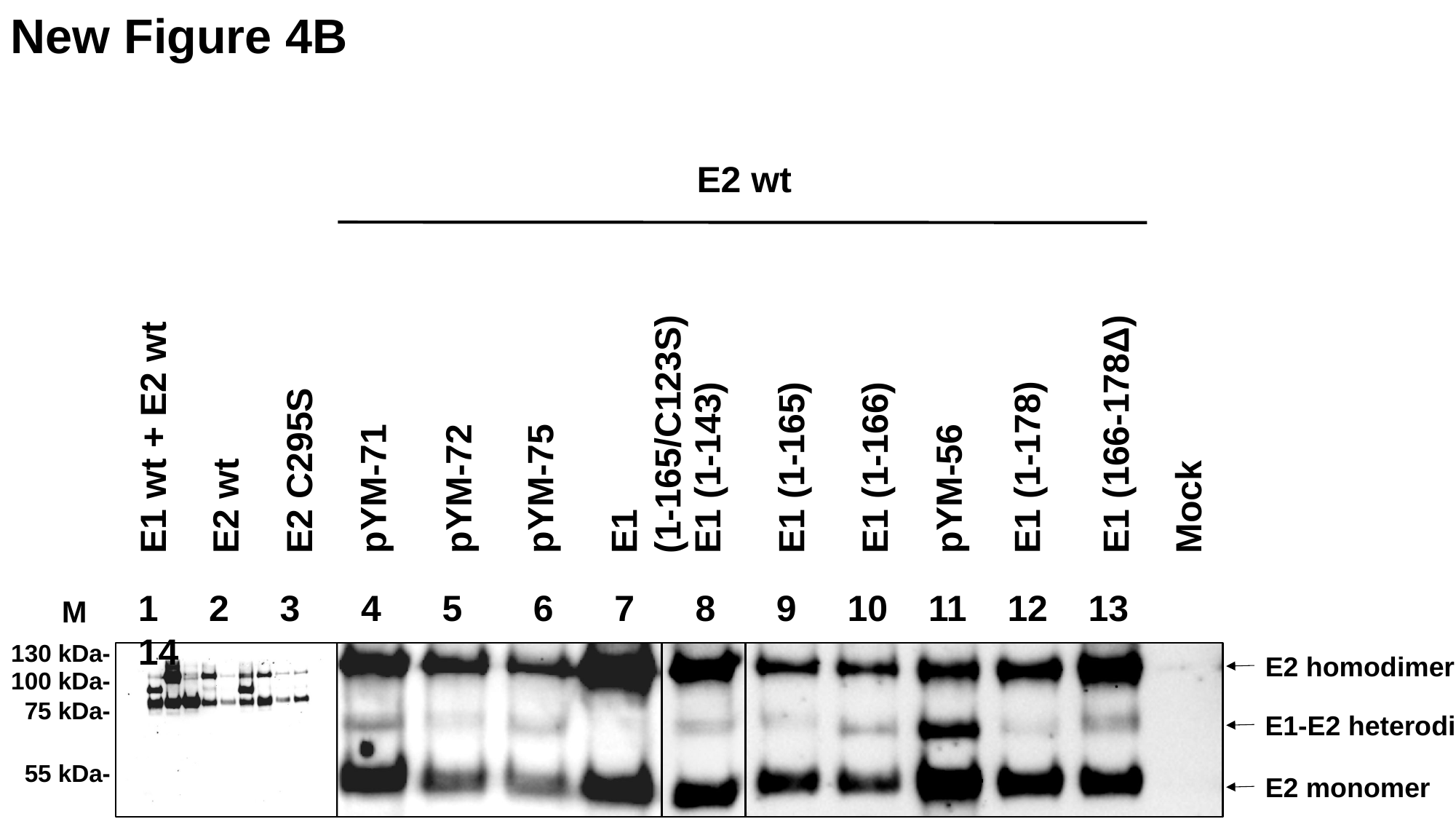

New Figure 4B
 E2 wt
E1 (1-165/C123S)
E1 (166-178Δ)
E1 wt + E2 wt
E2 C295S
E1 (1-178)
E1 (1-143)
E1 (1-165)
E1 (1-166)
pYM-71
pYM-72
pYM-75
pYM-56
E2 wt
Mock
1 2 3 4 5 6 7 8 9 10 11 12 13 14
M
130 kDa-
100 kDa-
75 kDa-
55 kDa-
E2 homodimer
E1-E2 heterodimer
E2 monomer

## Slide 12
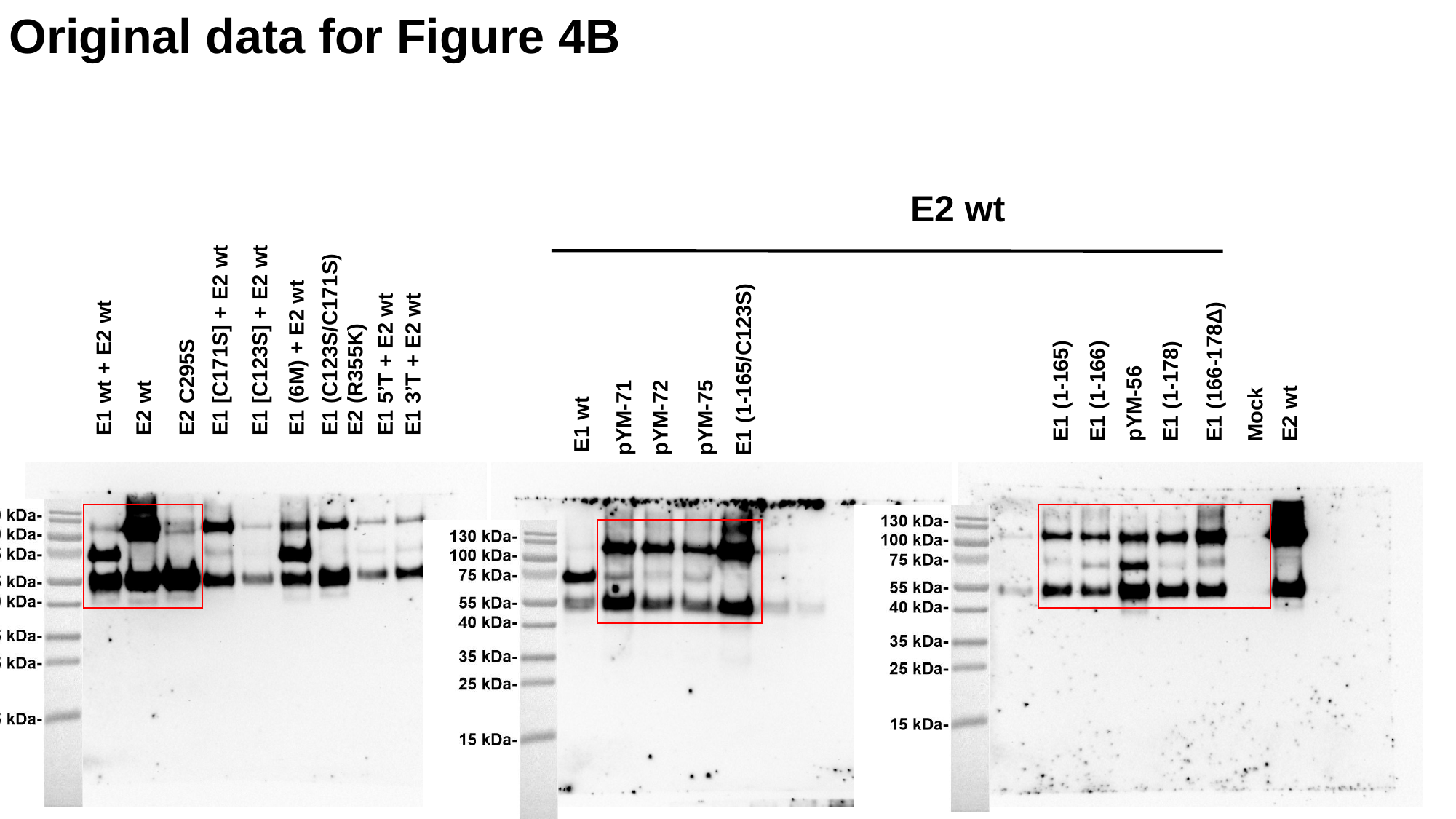

Original data for Figure 4B
 E2 wt
E1 (C123S/C171S)
E2 (R355K)
E1 [C171S] + E2 wt
E1 [C123S] + E2 wt
E1 (1-178)
E1 (6M) + E2 wt
E1 5’T + E2 wt
E1 3’T + E2 wt
E1 (1-165)
E1 (1-166)
E1 (1-165/C123S)
E1 (166-178Δ)
E1 wt + E2 wt
pYM-56
E2 wt
E2 C295S
E1 wt
pYM-71
pYM-72
pYM-75
Mock
E2 wt

## Slide 13
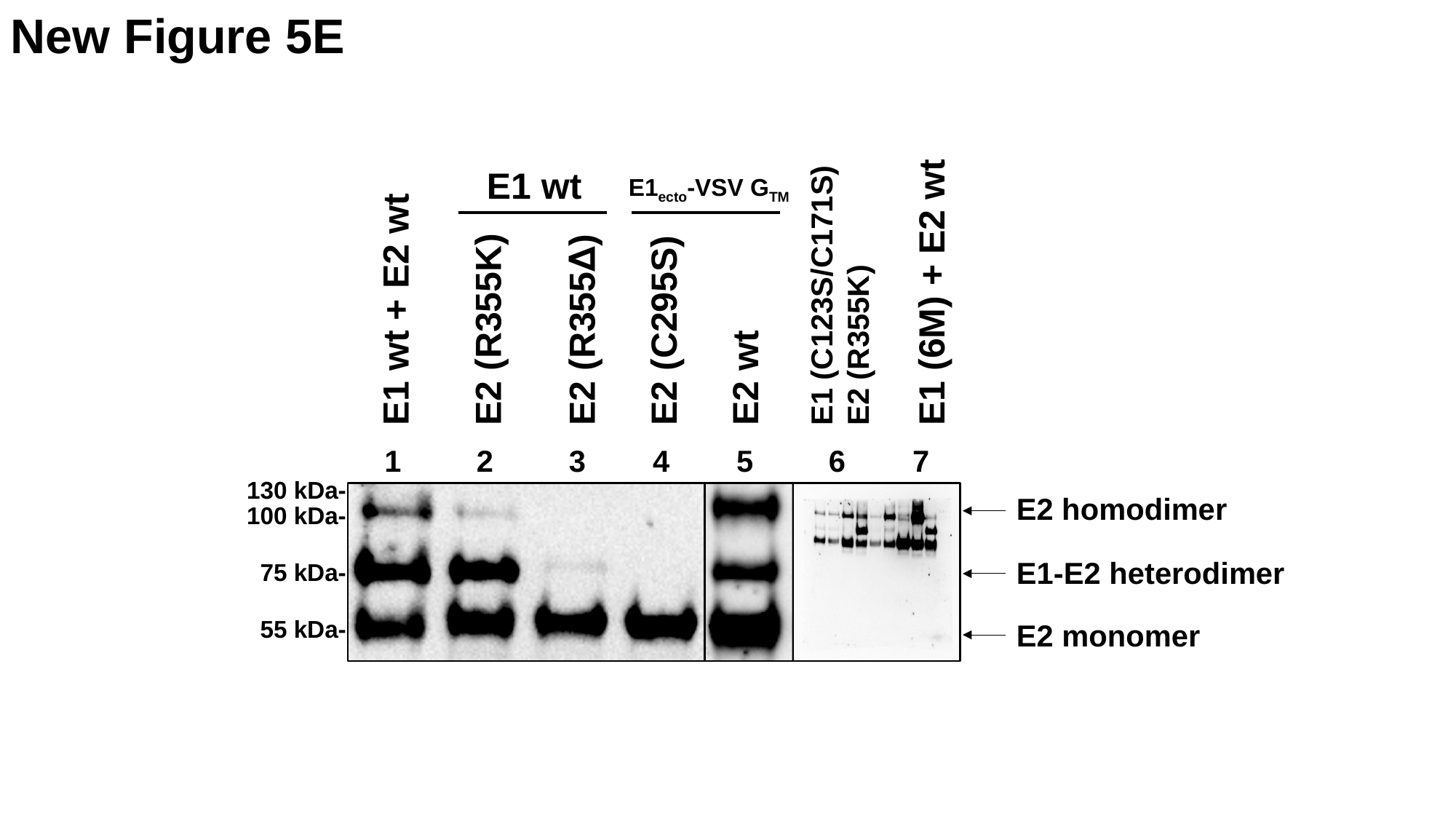

New Figure 5E
E1 (C123S/C171S)
E2 (R355K)
E1 (6M) + E2 wt
E1 wt + E2 wt
E2 (R355K)
E2 (R355∆)
E2 (C295S)
E2 wt
 E1 wt
 E1ecto-VSV GTM
 1 2 3 4 5 6 7
130 kDa-
100 kDa-
75 kDa-
55 kDa-
E2 homodimer
E1-E2 heterodimer
E2 monomer

## Slide 14
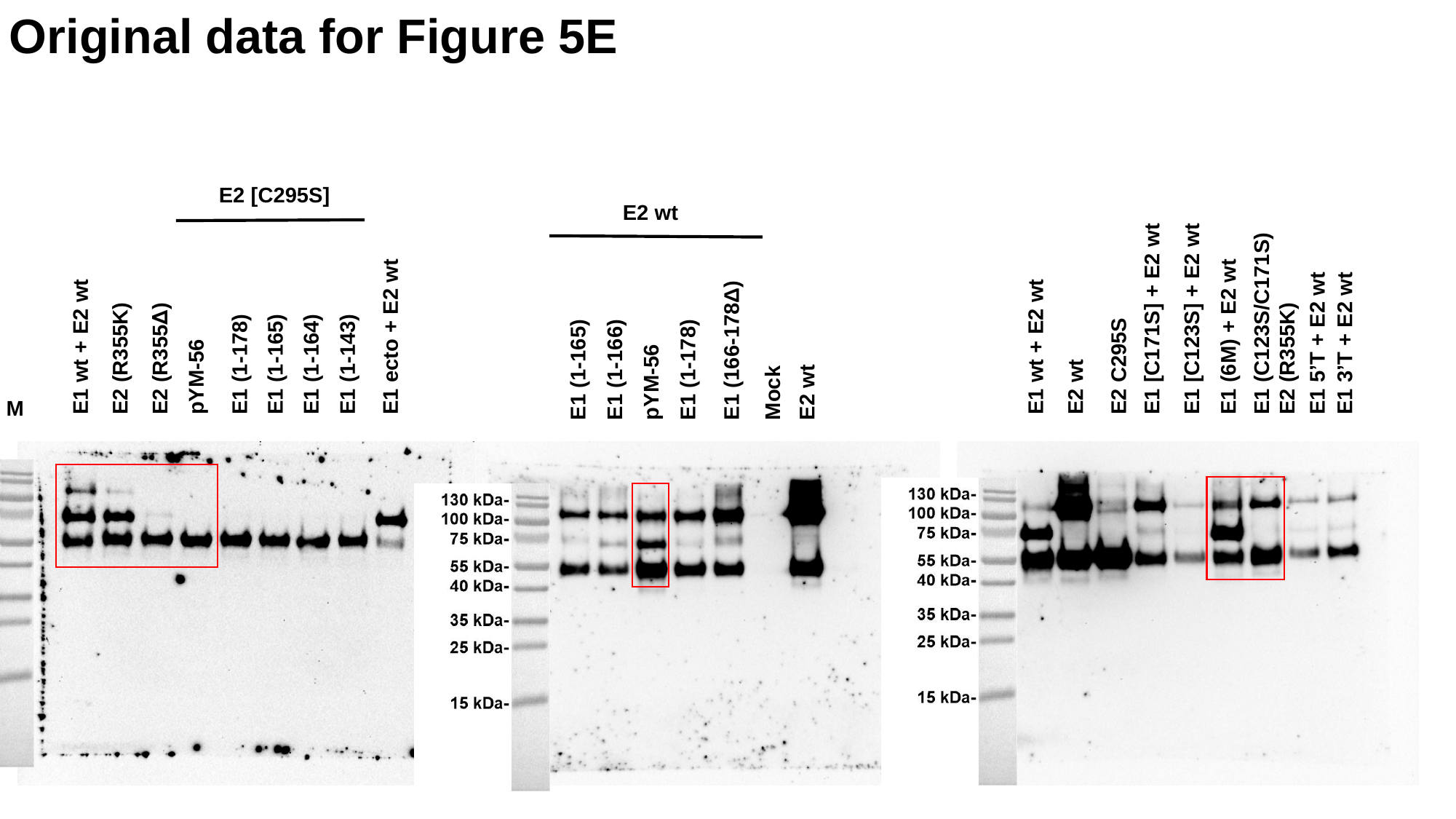

Original data for Figure 5E
E2 [C295S]
E2 wt
E1 (C123S/C171S)
E2 (R355K)
E1 [C171S] + E2 wt
E1 [C123S] + E2 wt
E1 (6M) + E2 wt
E1 5’T + E2 wt
E1 3’T + E2 wt
E1 wt + E2 wt
E2 wt
E2 C295S
E1 (1-178)
E1 (1-165)
E1 (1-166)
E1 (166-178Δ)
pYM-56
Mock
E2 wt
E1 ecto + E2 wt
E1 wt + E2 wt
E2 (R355K)
E2 (R355∆)
pYM-56
E1 (1-178)
E1 (1-165)
E1 (1-164)
E1 (1-143)
M
