# Supplementary material for: Interaction of Pestiviral E1 and E2 Sequences in Dimer Formation and Intracellular Retention
Source: Int J Mol Sci. 2021 Jul 6;22(14):7285. doi: 10.3390/ijms22147285 (PMC8306095; doi:10.3390/ijms22147285)
Supplement: Supplementary file 1 [file ijms-22-07285-s001.zip › ijms-1266080-suppl/ijms-1266080-suppl-submitted.pdf]

CLUSTAL O(1.2.4) multiple sequence alignment

```
E1|KJ950914.1|Pestivirus_J_NrPV/NYC-D23               NKIYCEKFHVLGDLVYVNSCLPMGLPTGARFVSKNVISLEPEKTAQIIPRLTHHLDSGIL         60
E1|EF100713.2|Porcine_Bungowannah                     -SPYCPVAKRVFNIIYTNNCTPLGLPDKSKIIGPGTFDISGR-DEFIFPKLPYHVDDFIL         58
E1|AY781152.3|Pronghorn_antelope_pestivirus           SEVYCKVEKRVGSLWYTRNCTPACLPGHTEILGAGVFDTNPQ-GRSLIPRLPGHITEAVI         59
E1|FJ527854.1|BVDV_XJ-04                              ASPYCDVERKIGYIWYTKNCTPACLPRNTRIIGPGKFDTNAE-DGKILREMGGHLSEFVL         59
E1|LC006970.1|BVDV_2_KZ-91-CP                         ASPYCDVERKIGYIWYTKNCTPACLPRNTKIIGPGKFDTNAE-DGKILHEMGGHLSEFVL         59
E1|GQ888686.2|BVDV_2_JZ05-1                           ASPYCEVERKIGYIWYTKNCTPACLPRNTKIIGPGKFDTNAE-DGKILHEMGGHLSEFAL         59
E1|KX096718.1|BVDV_2_HB-1511                          ASPYCDVERKIGYIWYTKNCTPACLPRNTRIIGPGKFDTNAE-DGKILHEMGGHLSEFVL         59
E1|JF714967.1|BVDV_2_HLJ-10                           ASPYCNVERKIGYIWYTKNCTPACLPRNTRIIGPGKFDTNAE-DGKILHEMGGHLSEFVL         59
E1|KT875169.1|BVDV_2_91W                              ASPYCDVERKIGYIWYTKNCTPACLPRNTRIIGPGKFDTNAE-DGKILHEMGGHLSEFAL         59
E1|HQ258810.1|BVDV_SH-28                              ASPYCDVERKIGYIWYTKNCTPACLPRNTRIIGPGKFDTNAE-DGKILHEMGGHLSEFVL         59
E1|KT832818.1|BVDV_2_USMARC-60765                     ASPYCDVERKIGYIWYTKNCTPACLPKNTRIIGPGKFDTNAE-DGKILHEMGGHLSEFVL         59
E1|AB567658.1|BVDV_Hokudai-Lab/09                     ASPYCDVEKKIGYVWYTKNCTPACLPKNTKIIGPGKFDTNAE-DGKIIHEMGGHLSEFVL         59
E1|KJ000672.1|BVDV_2_SD1301                           ASPYCNVEKKIGYIWYTRNCTPACLPKNTKIIGPGKFDTNAE-DGKIIHEMGGHLSEFAL         59
E1|JQ799141.1|BVDV_1_sichuan                          ASPYCNVERKLGYVWYTKNCTPACLPKNTKIIGPGMFDTNAE-DGKILHEMGGHLSEFIL         59
E1|AB078950.1|BVDV_1_KS86-1ncp                        ASPYCEVKRKIGYIWYTQNCTPACLPKNTKIIGPGKFDTNAD-DGKILYEMGGHLSEVLL         59
E1|JN400273.1|BVDV_1_SD0803                           ASPYCAVERKIGYIWFTKNCTPACLPKNTRIVGPGKFDTNAD-DGKILHEMGGHLSEVLL         59
E1|KX987157.1|BVDV_1_SLO/1170/2000                    ASPYCDVERKIGYIWYTKNCTPACLPRNTKIVGPGKFDTNGD-DGKILHEMGGHLSEVLL         59
E1|LT631725.1|BVDV_1_UM/126/07                        ASPYCNVERKIGYIWFTKNCTPACLPKNTKIVGPGKFDTNAD-DGKILHEMGGHLSEVLL         59
E1|KF896608.1|BVDV_1_Bega-like                        ASPYCDVERKLGYIWFTKNCTPACLPKNTKIVGPGMFDTNGD-DDKILHEMGGHLSEVLL         59
E1|M96751.1|BVDV_1_SD1                                ASPYCDVDRKIGYIWFTKNCTPACLPKNTKIIGPGKFDTNAE-DGKILHEMGGHLSEVLL         59
E1|KC757383.1|BVDV_1_10JJ-SKR                         SSPYCDVERKLGYIWFTKNCTPACLPKNTKIVGPGKFDTNAE-DGKIMHEMGGHLSEVLL         59
E1|AF526381.3|BVDV_1_ZM-95                            ASPYCEVERKIGYIWYTKNCTPACLPKNTKIVGPGRFDTNAE-DGKILHEMGGHLSEVLL         59
E1|KP941591.1|BVDV_1_USMARC-55925                     ASPYCEVERRLGYVWYTKNCTPACLPRNTKIIGPGRFDTNAE-DGKILHEMGGHLSEVLL         59
E1|LC089876.1|BVDV_1_Shitara/02/06                    ASPYCDVDRKVGYIWYTKNCTPACLPKNTMIIGPGKFDTNAE-DGKILHEMGGHLSEMLL         59
E1|KC853441.1|BVDV_1_SuwaCp                           ATPYCEVEQKIGYIWITKNCTPACLPKNTKIVGPGKFDTNAE-DGKILHEMGGHLSEVLL         59
E1|KX577637.1|BVDV_1_SLO/2407/2006                    ATPYCEVDRKLGYIWYTKNCTPACLPGNTKIVGPGKFDTNAE-DGKIIHEMGGHVSEVLL         59
E1|KP313732.1|BVDV_1_Carlito                          ATPYCEVERKLGYVWYTKNCTPACLPGNTKIVGPGKFDTNAE-DGKILHEMGGHLSEVLL         59
E1|FJ040215.1|BVDV_3_Th/04_KhonKaen                   ATPYCNVSRKIGYVWYTNNCTPACLPMNTRIVGPGKFDTNGD-DGKILHEMGGHLSELAI         59
E1|KC297709.1|BVDV_3_LVRI/cont-1                      ATPYCNINKRIGYVWYTNNCTPACLPKNPRIVGPGKFDTNGD-DGKILHEMGSHLSELTI         59
E1|KC788748.1|BVDV_3_Italy-129/07                     ATPYCNINKKIGYVWYTNNCTPACLPKNTRIVGPGKFDTNGD-DGKILHEMGSHLSELAI         59
E1|JX469119.1|BVDV_3_JS12/01                          ATPYCNINKKIGYVWYTNNCTPACLPKNTRIMGPGKFDTNGE-DGKILHEMGSHLSELAI         59
E1|JX985409.1|BVDV_3_CH-KaHo/cont                     ATPYCNINKKIGYVWYTNNCTPACLPKNTRIVGPGKFDTNGD-DGKILHEMGSHLSELAI         59
E1|AB871953.1|BVDV_3_D32/00_'HoBi'like                ATPYCNINKKIGYVWYTNNCTPACLPKNTRIVGPGKFDTNGD-DGKILHEMGSHLSELAI         59
E1|HQ231763.1|BVDV_3_Italy-1/10-1                     ATPYCNINKKIGYVWYTNNCTPACLPKNTRIVGPGKFDTNGD-DGKILHEMGSHLSELAI         59
E1|JQ612704.1|BVDV_3_Italy-83/10-ncp                  ATPYCNINKKIGYVWYTNNCTPACLPKNTRIVGPGKFDTNGD-DGKILHEMGSHLSELAI         59
E1|NC_018713.1|BVDV_3_LV03/12                         ATPYCNINKKIGYVWYTNNCTPACLPKNTRIVGPGKFDTNGD-DGKILHEMGSHLSELAI         59
E1|KJ660072.1|Pestivirus_PG-2                         -SPYCEVNRKLGYIWYTNNCTPACLPGNTKIIGPGKFDTNAE-DGKILHELGGHISEFLL         58
E1|MH410816.1|Pestivirus_PG-2_GIRAFFE                 -SPYCEVNRKLGYIWYTNNCTPACLPGNTKIIGPGKFDTNAE-DGKILHELGGHISEFLL         58
E1|AF144617.2|Pestivirus_giraffe-1_H138               ASPYCEVNKKLGYIWYTNNCTPACLPGNTKIIGPGKFDTNAE-DGKILHELGGHLSEFIL         59
E1|KJ463422.1|BDV_FNK2012-1                           QSPYCNVTRKIGYIWYTNNCTPACLPRNTKIIGPGKFDTNAE-DGKILHEMKGHISEFIL         59
E1|U70263.1|BDV_BD31                                  QSPYCNVTRKIGYIWYTNNCTPACLPKNTKIIGPGKFDTNAE-DGKILHEMKGHISEFIL         59
E1|KF925348.1|BDV_Coos_Bay-5_nc                       QSPYCNVTRKIGYIWYTNNCTPACLPKNTKIIGPGKFDTNAE-DGKILHEMKGHISEFIL         59
E1|AB897785.1|BDV_X818                                QSPYCNVTRKIGYVWYTNNCTPACLPKNTKIIGPGKFDTNAE-DGKILHEMRGHISEFIL         59
E1|AF144618.2|Pestivirus_reindeer-1_V60-Krefeld       QSPYCNVTKKLGYIWYTNNCTPACLPRNTKIVGPGKFDTNAE-DGKILHEMRGHISEFLL         59
E1|KC963426.1|BDV_JSLS12-01                           QSPYCNVTRKIGYIWYTNNCTPACLPGNTKIIGPGKFDTNAE-DGKILHEMRGHISEFLL         59
E1|AF407339.1|BDV_Aveyron                             QSPYCNVTKKLGYIWYTNNCTPACLPKNTKIIGPGKFDTNAE-DGKILHEMRGHISEFLL         59
E1|AY646427.1|CSFV_94.4/IL/94/TWN                     MSPYCNVTRKVGYIWYNNNCTPACLPKNTKIIGPGKFDTNAE-DGKILHEMGGHLSEFLL         59
E1|KP233070.1|CSFV_GXF29/2013                         MSPYCNVTRKIGYIWYTNNCTPACLPKNTKIIGPGKFDTNAE-DGKILHEMGGHLSEFLL         59
E1|KC851953.1|CSFV_IND/UK/LAL-290                     MSPYCNVTRKIGYIWYTNNCTPACLPKNTKIIGPGKFDTNAE-DGKILHEMGGHLSEFLL         59
E1|AF407339.1|CSFV_39                                 MSPYRNVTRKIGYIWYTNNCTPACLPRNTKIIGPGKFDTNAE-DGKILHEMGGHLSEFLL         59
E1|FJ529205.1|CSFV_Zj0801                             MSPYCNVTRKIGYIWYTNNCTPACLPTNTKIIGPGKFDTNAE-DGKILHEMGGHLSEFLL         59
E1|KJ619377.1|CSFV_Bergen                             MSPYCNVTRKIGYIWYTNNCTPACLPKNTKIIGPGKFDTNAE-DGKILHEMGGHLSEFLL         59
E1|KU504339.1|CSFV_GD19/2011                          MSPYCNVTRKVGYIWYTNNCTPACLPKNTKIIGPGKFDTNAE-DGKILHEMGGHLSEFLL         59
E1|KM362426.1|CSFV_IND/AS/GHY/G4                      MSPYCNVTRKIGYIWYTNNCTPACLPKNTKIIGPGKFDTNAE-DGKILHEMGGHLSEFLL         59
E1|GQ923951.1|CSFV_SXCDK                              MSPYCNVTRKIGYIWYTNNCTPACLPKNTKIIGPGKFDTNAE-DGKILHEMGGHLSEFLL         59
E1|J04358.2|CSFV_Alfort/Tuebingen                     MSPYCNVTRKIGYIWYTNNCTPACLPKNTKIIGPGKFDTNAE-DGKILHEMGGHLSEFLL         59
E1|AY259122|CSFV_Alfort/Tuebingen                     MSPYCNVTRKIGYIWYTNNCTPACLPKNTKIIGPGKFDTNAE-DGKILHEMGGHLSEFLL         59
E1|KF669877.1|CSFV_JJ9811                             LSPYCNVTSKIGYIWYTNNCTPACLPKNTRIIGPGKFDTNAE-DGKILHEMGGHLSEFLL         59
E1|X87939.1|CSFV_Alfort/187                           LSPYCNVTSKIGYIWYTNNCTPACLPKNTKIIGPGKFDTNAE-DGKILHEMGGHLSEFLL         59
E1|X87939|CSFV_Alfort/187                             LSPYCNVTSKIGYIWYTNNCTPACLPKNTKIIGPGKFDTNAE-DGKILHEMGGHLSEFLL         59
E1|KJ660072.1|CSFV_Riems                              LSPYCNVTSKIGYIWYTNNCTPACLPKNTKIIGPGKFDTNAE-DGKILHEMGGHLSEFLL         59
E1|GU270877.1|BDV_H2121_Chamois-1                     QSPYCNVTKKIGYVWYTSNCTPACLPKNTKIIGPGKFDTNAE-DGKILHEMKGHVSEFLL         59
E1|KF918753.1|BDV_Gifhorn_genotype-3                  QSPYCNVTRKIGYIWYTNNCTPACLPGNTKIIGPGKFDTNAE-DGKILHEMRGHISEFLL         59
E1|JX428945.1|Pestivirus_Aydin/04-TR                  QSPYCNVTRRIGYIWYTNNCTPACLPRNTKIIGPGKFDTNAE-DGKILHEMGGHISEFLL         59
E1|AF037405.1|Pestivirus_Aydin/04-TR                  QSPYCNVTRRIGYIWYTNNCTPACLPRNTKIIGPGKFDTNAE-DGKILHEMGGHISEFLL         59
E1|KM408491.1|Pestivirus_Burdur/05-TR                 QSPYCNVTRKIGYIWYTNNCTPACLPKNTKIIGPGKFDTNAE-DGKILHEMGGHISEFLL         59
                                                         *      :  :    .* *  **    ::. . :. .      :: .:  *: .  :

E1|KJ950914.1|Pestivirus_J_NrPV/NYC-D23               LVLVAMSDFMPETSSALYLILHFMIPNSRHRTISEEGLTMALNLTSTEPVSSVIPTSVYV         120
E1|EF100713.2|Porcine_Bungowannah                     LSLIAMSDFAPETSSIIYLALHYLMPSNDNRDFVMDLDPNKLNLTATKSVASVVPTSVNV         118
E1|AY781152.3|Pronghorn_antelope_pestivirus           LSLVALSEVMPETSSALYIALHYFLHPMN--ETIGYCDKNQLNLTITTTVDKVIPNSVYV         117
E1|FJ527854.1|BVDV_XJ-04                              LSLVVLSDFAPETASAIYLVLHFAIPQSH--ISVDTCDKNQLNITVAITVAEVIPGSVWN         117
E1|LC006970.1|BVDV_2_KZ-91-CP                         LSLVVLSDFAPETASVIYLVLHFTIPQSY--VSVDTCDKNQLNLTVATTVAEVIPGSVWN         117
E1|GQ888686.2|BVDV_2_JZ05-1                           LSLVVLSDFAPETASVIYLVLHFAIPQSH--INVDTCDKSQLNLTVATTVAEVIPGTVWN         117
E1|KX096718.1|BVDV_2_HB-1511                          LSLVVLSDFAPETSSAIYLVLHFAIPQSH--VNVDTCDKNQLNLTVATTVAEVIPGSVWN         117
```

```
E1|JF714967.1|BVDV_2_HLJ-10                         LSLVVLSDFAPETASVIYLVLHFAIPQSH--VSVDTCDKNQLNLTVATTVAEVIPGTVWN      117
E1|KT875169.1|BVDV_2_91W                            LSLVVLSDFAPETASAIYLVLHFVIPQSH--VSVDTCDKNQLNLTVATTVAEVIPGTVWN      117
E1|HQ258810.1|BVDV_SH-28                            LSLVVLSDFAPETASVIYLVLHFAIPQNH--VNVDTCDKNQLNLTVATTVAEVIPGSVWN      117
E1|KT832818.1|BVDV_2_USMARC-60765                   LSLVVLSDFAPETASVIYLVLHFAIPQNH--INVDTCDKNQLNLTVATTVAEVIPGSVWN      117
E1|AB567658.1|BVDV_Hokudai-Lab/09                   LSLVVLSDFAPESASVIYLVLHYAIPQNH--AEVTTCDKNQLNLTVTTTTADVIPGSVWN      117
E1|KJ000672.1|BVDV_2_SD1301                         LSLVVLSDFSPETASVIYLVLHYVIPQNH--VEVTTCDKNQLNLTIATTTADVIPGSVWN      117
E1|JQ799141.1|BVDV_1_sichuan                        LSLVVLSDFAPETASALYLVLHFTIPQSH--TPVMDCDKSQLNLTVGLTTADVVPSSVWN      117
E1|AB078950.1|BVDV_1_KS86-1ncp                      LSLVVMSDFAPETASAIYLILHFSIPQSH--TEVSDCDKSQLNLTVGLTTADVIPGSVWN      117
E1|JN400273.1|BVDV_1_SD0803                         MSLVVLSDFAPETASVVYLILHFSIPQGH--TDVLDCDKGQLNLTVGLRTEDVIPGSVWN      117
E1|KX987157.1|BVDV_1_SLO/1170/2000                  LSLVVLSDFAPETASAVYLILHFSIPQSH--SDILDCDKNQLNLTVELTTEQVIPGSVWN      117
E1|LT631725.1|BVDV_1_UM/126/07                      LSLVVLSDFAPETASVMYIILHFFIPQSH--IDVSDCDKNQLNLTIGLKTDDVVPNSVWN      117
E1|KF896608.1|BVDV_1_Bega-like                      LSLVVLSDFAPETASAMYLILHFSIPQSH--TDITDCDKNQLNLTIGLTTADMVPGSVWN      117
E1|M96751.1|BVDV_1_SD1                              LSLVVLSDFAPETASAMYLILHFSIPQSH--VDITDCDKTQLNLTIELTTADVIPGSVWN      117
E1|KC757383.1|BVDV_1_10JJ-SKR                       LSLVVLSDFAPETASTLYLVLHFSIPQRH--TDILDCDKSQLNLTVGVTTADVIPGSVWN      117
E1|AF526381.3|BVDV_1_ZM-95                          LSLVVLSDFAPETASVAYLALHFGIPQRH--TEVLDCDKNQLNLTVGVTTEDVIPGSVWN      117
E1|KP941591.1|BVDV_1_USMARC-55925                   LSVVVLSDFAPETASLIYLILHFSIPQGH--TDIHDCDRNQLNLTVELTTAEVIPGSVWN      117
E1|LC089876.1|BVDV_1_Shitara/02/06                  LSLVVMSDFAPETASVIYLILHFSIPQGH--TEVLDCDKNQLNLTVGLTTADVIPGSVWN      117
E1|KC853441.1|BVDV_1_SuwaCp                         LSLVVLSDFAPETASVIYLVLHFTVPQSH--TDVLDCDKNQLNLTIGVTTADVIPGSVWN      117
E1|KX577637.1|BVDV_1_SLO/2407/2006                  LSLVVLSDFAPETASVVYLILHFSIPQRH--TDILDCDKNQLNLTVELTTADVIPGSVWN      117
E1|KP313732.1|BVDV_1_Carlito                        LSLVVLSDFAPETASVMYLVLHFSIPQSH--TDILDCDKDQLNLTVKLTTADVIPGSVWN      117
E1|FJ040215.1|BVDV_3_Th/04_KhonKaen                 LALVVMSDFAPESASVLYLILHFSIPQAH--EEVDQCDKNQLNLTVGLRTDEVVPSSVWN      117
E1|KC297709.1|BVDV_3_LVRI/cont-1                    LALVVMSDFAPESASVLYLILHFSIPQAH--EEVDQCDRNQLNLTVSLRTDEVVPSSVWN      117
E1|KC788748.1|BVDV_3_Italy-129/07                   LALVVMSDFTPESTSVLYLILHFSIPQAH--EEVDQCDRNQLNLTVSLRTDEVVPSSVWN      117
E1|JX469119.1|BVDV_3_JS12/01                        LALVVMSDFAPESASVLYLILHFSIPQAH--EEVDQCDRNQLNLTVSLRTDEVVPSSVWN      117
E1|JX985409.1|BVDV_3_CH-KaHo/cont                   LALVVMSDFAPESASVLYLILHFSIPQAH--EEVDQCDRNQLNLTVSLRTDEVVPSSVWN      117
E1|AB871953.1|BVDV_3_D32/00_'HoBi'like              LALVVMSDFAPESASVLYLILHFSIPQAH--EEVDQCDRNQLNLTVGLRTDEVVPSSVWN      117
E1|HQ231763.1|BVDV_3_Italy-1/10-1                   LALVVMSDFAPESASVLYLILHFSIPQAH--EEVDQCDRNQLNLTVSLRTDEVVPSSVWN      117
E1|JQ612704.1|BVDV_3_Italy-83/10-ncp                LALVVMSDFAPESASVLYLILHFSIPQAH--EEVDQCDRNQLNLTVSLRTDEVVPSSVWN      117
E1|NC_018713.1|BVDV_3_LV03/12                       LALVVMSDFAPESASVLYLILHFSIPQAH--EEVDQCDRNQLNLTVSLRTDEVVPSSVWN      117
E1|KJ660072.1|Pestivirus_PG-2                       LSLVVLSDFAPETASAIYLVLHYTIPQKH--EVVENCDMNQLNLTVTKRVKDVIPSSVWN      116
E1|MH410816.1|Pestivirus_PG-2_GIRAFFE               LSLVVLSDFAPETASAIYLVLHYTIPQKH--EVVENCDMNQLNLTVTKRVKDVIPSSVWN      116
E1|AF144617.2|Pestivirus_giraffe-1_H138             LSLVVLSDFAPETASAIYLVLHYTMPQKY--EVVGSCDRNQLNLTVKTRVEDVIPSSVWN      117
E1|KJ463422.1|BDV_FNK2012-1                         LSLVVLSDFAPETASTLYLVLHFTLPQTY--EVPSGCDTNQLNLTVDLRVDDVIPSSVWN      117
E1|U70263.1|BDV_BD31                                LSLVVLSDFAPETASTLYLVLHFTLPQTY--EVPSECDTNQLNLTVGLRVDDVIPSSVWN      117
E1|KF925348.1|BDV_Coos_Bay-5_nc                     LSLVVLSDFAPETASTLYLVLHFTLPQTY--EVPNECDTSQLNLTVGLRVDDVIPSSVWN      117
E1|AB897785.1|BDV_X818                              LSLVVLSDFAPETASTLYLVLHFALPQTH--EVPSVCDTNQLNLTVSLRVDDVIPSSVWN      117
E1|AF144618.2|Pestivirus_reindeer-1_V60-Krefeld     LSLVVLSDFAPETASTLYLILHFTIPQSH--EAPSECDTNQLNLTIGLRVDEVVPSSVWN      117
E1|KC963426.1|BDV_JSLS12-01                         LSLVILSDFAPETASSLYLILHFAIPQSH--EIPDGCDTNQLNLTVNLRVEDVIPSSVWN      117
E1|AF407339.1|BDV_Aveyron                           LSLVVLSDFAPETASALYLLLHFAIPQSH--ENPSSCDTNQLNLTTGLKVEDVIPSSVWN      117
E1|AY646427.1|CSFV_94.4/IL/94/TWN                   LSLVVLSDFAPETASALYLIFHYTIPQIH--EVPEDCDTNQLNLTVELRTEDVVPSSVWN      117
E1|KP233070.1|CSFV_GXF29/2013                       LSLVILSDFAPETASTLYLILHYAVPQSY--GEPEGCDTNQLNLTVGLRTQDVVPSSVWN      117
E1|KC851953.1|CSFV_IND/UK/LAL-290                   LSLVILSDFAPETASTLYLILHYAIPQSH--EEPESCDTNQLNLTVGLKTEDVVPSSVWN      117
E1|AF407339.1|CSFV_39                               LSLVILSDFAPETASTLYLILHYAIPQSH--EEPEGCDTNQLNLTVGLKTEDVVPSSVWN      117
E1|FJ529205.1|CSFV_Zj0801                           LSLVILSDFAPETASTLYLILHYAIPQSH--EEPEGCDTNQLNLTVGLRTEDVVPSSVWN      117
E1|KJ619377.1|CSFV_Bergen                           LSLVILSDFAPETASTLYLILHYAIPQSH--DEPEGCDTNQLNLTVGLRTEDVVPSSVWN      117
E1|KU504339.1|CSFV_GD19/2011                        LSLVILSDFAPETASTLYLILHYAIPQSH--EEPEGCDTNQLNLTVGLRTEDVVPSSVWN      117
E1|KM362426.1|CSFV_IND/AS/GHY/G4                    LSLVILSDFAPETASTLYLILHYAIPQPH--KEPEGCDTNQLNLTVGLRTEDVVPSSVWN      117
E1|GQ923951.1|CSFV_SXCDK                            LSLVILSDFAPETASTLYLILHYAIPQSH--EEPEGCDTNQLNLTVGLRTEDVIPSSVWN      117
E1|J04358.2|CSFV_Alfort/Tuebingen                   LSLVILSDFAPETASTLYLILHYAIPQSH--EEPEGCDTNQLNLTVKLRTEDVVPSSVWN      117
E1|AY259122|CSFV_Alfort/Tuebingen                   LSLVILSDFAPETASTLYLILHYAIPQSH--EEPEGCDTNQLNLTVKLRTEDVVPSSVWN      117
E1|KF669877.1|CSFV_JJ9811                           LSLVVLSDFAPETASTLYLILHYAIPQVH--KEPDGCDTNQLNLTVELRTEDVVPSSVWN      117
E1|X87939.1|CSFV_Alfort/187                         LSLVVLSDFAPETASALYLILHYVIPQSH--EEPEGCDTNQLNLTVELRTEDVIPSSVWN      117
E1|X87939|CSFV_Alfort/187                           LSLVVLSDFAPETASALYLILHYVIPQSH--EEPEGCDTNQLNLTVELRTEDVIPSSVWN      117
E1|KJ660072.1|CSFV_Riems                            LSLVVLSDFAPETASALYLIFHYVIPQPH--DEPEGCDTNQLNLTVELRTEDVIPSSVWN      117
E1|GU270877.1|BDV_H2121_Chamois-1                   LSLVILSDFAPETASTLYLILHFTIPQSY--ESPDDCDTNQLNLTVSLKVEDVIPSSVWN      117
E1|KF918753.1|BDV_Gifhorn_genotype-3                LSLVVLSDFAPETASVLYLILHFSIPQGY--ESPDQCDMNQLNLTVDLKVEDVIPSSVWN      117
E1|JX428945.1|Pestivirus_Aydin/04-TR                LSLVVLSDFAPETASAIYLVLHFTIPQSY--ENPKDCDKNQLNLTIGLRTEDVVPSSVWN      117
E1|AF037405.1|Pestivirus_Aydin/04-TR                LSLVVLSDFAPETASAIYLVLHFTIPQSY--ENPKDCDKNQLNLTIGLRTEDVVPSSVWN      117
E1|KM408491.1|Pestivirus_Burdur/05-TR               LSLVVLSDFAPETASALYLILHFTIPQSY--ENPADCDKNQLNLTVGLKTEDVVPSSVWN      117
                                                    : :: :*:. **::*  *: :*: :                  **:*    . .::* :*

E1|KJ950914.1|Pestivirus_J_NrPV/NYC-D23             EGQWTCWKPSWWPYNADIALFFEGAFEMLELIARAVGDLMKVWTEATAVAFLCFLIKAFR      180
E1|EF100713.2|Porcine_Bungowannah                   LGEWVCVKPSWWPYSAEITNLIGGVITVADLVIKTIEELLNLWTEATAVAFLAALIKIFR      178
E1|AY781152.3|Pronghorn_antelope_pestivirus         LGQWVCVKPGWWPYDSEVTLVVNEVINVLDIGGRAARVLLQVWDAATAIAVLIFIMKVAR      177
E1|FJ527854.1|BVDV_XJ-04                            LGKYVCIRPNWWPYETTAVFVLEEAGQVVKLGLRAIRDLTRIWNAATTTAFLVFLVKVLR      177
E1|LC006970.1|BVDV_2_KZ-91-CP                       LGKYVCIRPDWWPYETATVFVLEEAGQVVKLGLRAIRDLTRIWNAATTTAFLVFLVKALR      177
E1|GQ888686.2|BVDV_2_JZ05-1                         LGKYVCIRPDWWPYETTTVFVLEEAGQVIKLGLRAIRDLTRIWNAATTTAFLVFLVKVLR      177
E1|KX096718.1|BVDV_2_HB-1511                        LGKYVCIRPDWWPYETTTVLVLEEAGQVVKLGLRAIRDLTRIWNAATTTAFLVFLVKVLR      177
E1|JF714967.1|BVDV_2_HLJ-10                         LGKYVCIRPDWWPYETTTVFVLEEAGQVVKLGLRAIRDLTRIWNAATTTAFLVFLVKVLR      177
E1|KT875169.1|BVDV_2_91W                            LGKYVCIRPDWWPYETTTVFVLEEAGQVVKLGLRAIRDLTRIWNAATTTAFLVFLVKVLR      177
E1|HQ258810.1|BVDV_SH-28                            LGKYVCIRPDWWPYETTTVFVLEEAGQVVKLGLRAIRDLTRIWNAATTIAFLVFLVGALR      177
E1|KT832818.1|BVDV_2_USMARC-60765                   LGRYVCIRPDWWPYETTTVFVLEEAGQVVKLGLRAIRDLTRIWNAATTTAFLVFLVKVLR      177
E1|AB567658.1|BVDV_Hokudai-Lab/09                   LGKYVCIRPDWWPYETATVFALEEAGQVVKLGLRAIRDLTRIWNAATTTAFLVFLVKVLR      177
E1|KJ000672.1|BVDV_2_SD1301                         LGKYVCIRPDWWPYETTVVFALEEAGQVVKLGMRAIRDLTRIWNAATTIAFLVFLVKVLR      177
E1|JQ799141.1|BVDV_1_sichuan                        LGKWVCVRPDWWPYETATVLLFEEAGQVVKIALRAVRDITRIWNAATTTAFLVCLVRVAR      177
E1|AB078950.1|BVDV_1_KS86-1ncp                      IGRYVCIRPDWWPYETTTVLVVEEVSQVVKLVLRAIRDLTRIWDAATSTAFLVCLVKVVR      177
E1|JN400273.1|BVDV_1_SD0803                         LGRYVCIRPNWWPYETTTVLAFEEIGQVIKIALRALRDLTRIWNAATATAFLVCLVKVVR      177
E1|KX987157.1|BVDV_1_SLO/1170/2000                  LGRYVCIRPNWWPYETAPVLALEEIGQVINIVLRALRDLTRIWNAATTTAFLVCLVKVVR      177
E1|LT631725.1|BVDV_1_UM/126/07                      MGKYVCIRPNWWPYETTAVLAFEEVSQVVKIVLRALRDLTRIWNAATTTAFLVCLVKVVR      177
E1|KF896608.1|BVDV_1_Bega-like                      LGKYVCIRPNWWPYETVTVLAFEEVGQVIKIVLRALRDLTRIWNAATTTAFLVCLVKVVR      177
E1|M96751.1|BVDV_1_SD1                              LGKYVCIRPDWWPYETAAVLAFEEVGQVVKIVLRALRDLTRIWNAATTTAFLVCLIKMVR      177
```

```
E1|KC757383.1|BVDV_1_10JJ-SKR                       LGKWVCIRPDWWPYETAPVLAFEEAGQVVRIALRALRDLTRIWNAATTTAFLVCLVKVVR    177
E1|AF526381.3|BVDV_1_ZM-95                          LGRYVCIRPDWWPYETATVLVFEEISQVIKLVLRALRDLMRIWNAATTTAFLVCLVKVVR    177
E1|KP941591.1|BVDV_1_USMARC-55925                   LGKYVCIRPDWWPYETATVLVFEEVGQVIKIVLRALRDLTRIWTAATTTAFLVCLVKVVR    177
E1|LC089876.1|BVDV_1_Shitara/02/06                  LGRYVCIRPDWWPYETATVMAFEEVGQVIKIALRALKDLTRIWNAATTTAFLVCLVKVVR    177
E1|KC853441.1|BVDV_1_SuwaCp                         LGKYVCIRPDWWPYETAAVLVFEEVGQVIKIALRALRDLTRIWNAATTTAFLVCLVKVVR    177
E1|KX577637.1|BVDV_1_SLO/2407/2006                  LGKYVCIRPDWWPYETATVLAFEEIGQVIKIALRALRDLTRIWNAATTTAFLVCMVKVVR    177
E1|KP313732.1|BVDV_1_Carlito                        LGKYVCIRPDWWPYETATVLAFEEVGQVIKIALRALRDLTRIWNAATTTAFLVCLVKVVR    177
E1|FJ040215.1|BVDV_3_Th/04_KhonKaen                 LGKWVCVRPSWWPYETATVLAFEEIGQVLKLILRALKDLTNMWNAASTTAFLVCLVKILR    177
E1|KC297709.1|BVDV_3_LVRI/cont-1                    LGKWVCVRPPWWPYETATVLAFEEIGQVLKLILRALKDLTNMWNAASTTAFLVCLVKILR    177
E1|KC788748.1|BVDV_3_Italy-129/07                   LGKWVCVRPPWWPYETATVLAFEEIGQVLKLILRALKDLTNMWNAASTTAFLVCLVKILR    177
E1|JX469119.1|BVDV_3_JS12/01                        LGKWVCVRPSWWPYETATVLAFEEIGQVLKLILRALKDLTNMWNAASTTAFLVCLVKILR    177
E1|JX985409.1|BVDV_3_CH-KaHo/cont                   LGKWVCVRPSWWPYETATVLAFEEIGQVLKLILRALKDLTNMWNAASTTAFLVCLVKILR    177
E1|AB871953.1|BVDV_3_D32/00_'HoBi'like              LGKWVCVRPPWWPYETATVLAFEEIGQVLKLILRALKDLTNMWNAASTTAFLVCLVKILR    177
E1|HQ231763.1|BVDV_3_Italy-1/10-1                   LGKWVCVRPPWWPYETATVLAFEEIGQVLKLILRALKDLTNMWNAASTTAFLVCLVKILR    177
E1|JQ612704.1|BVDV_3_Italy-83/10-ncp                LGKWVCVRPPWWPYETATVLAFEEIGQVLKLILRALKDLTNMWNAASTTAFLVCLVKILR    177
E1|NC_018713.1|BVDV_3_LV03/12                       LGKWVCVRPPWWPYETATVLAFEEIGQVLKLILRALKDLTNMWNAASTTAFLVCLVKILR    177
E1|KJ660072.1|Pestivirus_PG-2                       IGKYVCVRPDWWPYETTTVLIFEEISHVVKLVLRALRDLTRIWNAASTTAFLICLVKVLR    176
E1|MH410816.1|Pestivirus_PG-2_GIRAFFE               IGKYVCVRPDWWPYETTTVLIFEEISHVVKLVLRALRDLTRIWNAASTTAFLICLVKVLR    176
E1|AF144617.2|Pestivirus_giraffe-1_H138             IGKYVCVRPDWWPYETTTVFIFEEVSQVVKLVLRALRDLTRIWNAASTTAFLICLVKVLR    177
E1|KJ463422.1|BDV_FNK2012-1                         LGKYVCVRPDWWPYETTIVLLFEEAGQVVKLVLRAIRDLTRIWNSASTTAFLICLIKVLR    177
E1|U70263.1|BDV_BD31                                LGKYVCVRPDWWPYETTIVLLFEEAEQVVKLVLRAIRDLTRVWNSASTTAFLICLAKVLR    177
E1|KF925348.1|BDV_Coos_Bay-5_nc                     LGKYVCVRPDWWPYETTIVLLFEEAEQVVKLVLRAIRDLTRVWNSASTTAFLICLVKVLR    177
E1|AB897785.1|BDV_X818                              LGKYVCVRPDWWPYETTMVLLFEEAGQVVKLVLRAIRDLTRVWNSASTTAFLICLVKVLR    177
E1|AF144618.2|Pestivirus_reindeer-1_V60-Krefeld     LGKYVCVRPDWWPYETTVVLLCEEAGQVIKLVLRAIRDLTRVWNSASTTAFLICLIKVLR    177
E1|KC963426.1|BDV_JSLS12-01                         LGKYVCVRPDWWPYETTAVLLFEEAGQIVKLILRAIRDLTRVWNSASTTAFLICLVKVLR    177
E1|AF407339.1|BDV_Aveyron                           LGKYVCVRPDWWPYETTVVLLFEEAGQVVKLILRAIRDLTRVWNSASTTAFLICLVKVLR    177
E1|AY646427.1|CSFV_94.4/IL/94/TWN                   VGEYVCVRPDWWPYETKVVLLFEEAGQVIKLALRAMRDLTRVWSSASTIAFLICLIKVLR    177
E1|KP233070.1|CSFV_GXF29/2013                       IGKYVCVRPDWWPYETKVALLFEEAGQVIKLALRALRDLTRVWNSASTTAFLICLIKVLR    177
E1|KC851953.1|CSFV_IND/UK/LAL-290                   IGKYVCVRPDWWPYETKVALLFEEVGQVIKLALRALRDLTRVWNSASTTAFLICLIKVLR    177
E1|AF407339.1|CSFV_39                               IGKYVCVRPDWWPYETKVALLFEEAGQVIKLALRALRDLTRVWNSASTTAFLICLIKILR    177
E1|FJ529205.1|CSFV_Zj0801                           IGKYVCVRPDWWPYETKVALLFEEAGQVVKLALRALRDLTRVWNSASTTAFLICLIKILR    177
E1|KJ619377.1|CSFV_Bergen                           IGKYVCVRPDWWPYETKVALLFEEAGQVIKLALRALRDLTRVWNSASTTAFLICLIKTLR    177
E1|KU504339.1|CSFV_GD19/2011                        IGKYVCVRPDWWPYETKVALLFEEAGQVIKLALRALRDLTRVWNSASTTAFLICLIKILR    177
E1|KM362426.1|CSFV_IND/AS/GHY/G4                    IGKYVCVRPDWWPYETKVALLFEEAGQVIKLVLRALRDLTRVWNSASTTAFLICLIKVLR    177
E1|GQ923951.1|CSFV_SXCDK                            IGKYVCVRPDWWPYETKVALLFEEAGQVVKLVLRALRDLTRVWNSASTTAFLICLIKVLR    177
E1|J04358.2|CSFV_Alfort/Tuebingen                   IGKYVCVRPDWWPYETKVALLFEEAGQVIKLVLRALRDLTRVWNSASTTAFLICLIKVLR    177
E1|AY259122|CSFV_Alfort/Tuebingen                   IGKYVCVRPDWWPYETKVALLFEEAGQVIKLVLRALRDLTRVWNSASTTAFLICLIKVLR    177
E1|KF669877.1|CSFV_JJ9811                           VGKYVCVRPDWWPYETKVALLFEEAGQVIKLALRALRDLTRVWNSASTTAFLICLIKVLR    177
E1|X87939.1|CSFV_Alfort/187                         VGKYVCVRPDWWPYETKVALLFEEAGQVVKLALRALRDLTRVWNSASTTAFLICLIKVLR    177
E1|X87939|CSFV_Alfort/187                           VGKYVCVRPDWWPYETKVALLFEEAGQVVKLALRALRDLTRVWNSASTTAFLICLIKVLR    177
E1|KJ660072.1|CSFV_Riems                            VGKYVCVRPDWWPYETEVALLFEEVGQVVKLALRALRDLTRVWNSASTIAFLICLIKVLR    177
E1|GU270877.1|BDV_H2121_Chamois-1                   LEKYVCIRPYWWPYETTVALLFEEAGQVLKLALRAIRDLTRVWNSASTTAFLICLVKLLR    177
E1|KF918753.1|BDV_Gifhorn_genotype-3                LGKYVCIRPDWWPYETTVVLLFEEAGQVIKLALRAIRDLTRVWSSASTTAFLICLVKVLR    177
E1|JX428945.1|Pestivirus_Aydin/04-TR                IGKYVCIRPDWWPYETTVVLLFEEVGQVIKLALRALRDLTRVWNSASTTAFLICLIKVLR    177
E1|AF037405.1|Pestivirus_Aydin/04-TR                IGKYVCIRPDWWPYETTVVLLFEEVGQVIKLALRALRDLTRVWNSASTTAFLICLIKVLR    177
E1|KM408491.1|Pestivirus_Burdur/05-TR               LGKYVCIRPDWWPYETTVVLLFEEVGQVIKLALRALRDLTRVWNSASTTAFLICLIKVLR    177
                                                      .:.* :* ****.:  .        :  :  ::   : .:*  *:: *.*  :    *

E1|KJ950914.1|Pestivirus_J_NrPV/NYC-D23             GQILQGVILLLLLSSAEG 198
E1|EF100713.2|Porcine_Bungowannah                   GQPIQAVAWLIIIGGAQA 196
E1|AY781152.3|Pronghorn_antelope_pestivirus         GQLIQGLIWLLLLTGTEA 195
E1|FJ527854.1|BVDV_XJ-04                            GQLIQGLLWLMLITGAQS 195
E1|LC006970.1|BVDV_2_KZ-91-CP                       GQLIQGLLWLMLITGAQG 195
E1|GQ888686.2|BVDV_2_JZ05-1                         GQLIQGLLWLMLITGAQG 195
E1|KX096718.1|BVDV_2_HB-1511                        GQLIQGLLWLMLITGAQS 195
E1|JF714967.1|BVDV_2_HLJ-10                         GQLIQGLLWLMLITGAQS 195
E1|KT875169.1|BVDV_2_91W                            GQLIQGLLWLMLITGAQS 195
E1|HQ258810.1|BVDV_SH-28                            GQLIQGLLWLMLITGAQG 195
E1|KT832818.1|BVDV_2_USMARC-60765                   GQLIQGLLWLMLITGAQS 195
E1|AB567658.1|BVDV_Hokudai-Lab/09                   GQLIQGLLWLMLITGAQG 195
E1|KJ000672.1|BVDV_2_SD1301                         GQLIQGLLWLMLITGVQS 195
E1|JQ799141.1|BVDV_1_sichuan                        GQVLQGLIWLLLITGAQG 195
E1|AB078950.1|BVDV_1_KS86-1ncp                      GQVLQGILWLLLISGVQG 195
E1|JN400273.1|BVDV_1_SD0803                         GQVLQGILWLLLITGVQG 195
E1|KX987157.1|BVDV_1_SLO/1170/2000                  GQVLQGILWLLLITGVQG 195
E1|LT631725.1|BVDV_1_UM/126/07                      GQVLQGILWLLLITGVQG 195
E1|KF896608.1|BVDV_1_Bega-like                      GQVLQGILWLLLITGVQG 195
E1|M96751.1|BVDV_1_SD1                              GQVVQGILWLLLITGVQG 195
E1|KC757383.1|BVDV_1_10JJ-SKR                       GQVVQGVIWLLLITGVQG 195
E1|AF526381.3|BVDV_1_ZM-95                          GQVLQGVLWLLLITGAQG 195
E1|KP941591.1|BVDV_1_USMARC-55925                   GQVLQGILWLILITGAQG 195
E1|LC089876.1|BVDV_1_Shitara/02/06                  GQVLQGILWLLLITGVQG 195
E1|KC853441.1|BVDV_1_SuwaCp                         GQILQGILWLLLITGVQG 195
E1|KX577637.1|BVDV_1_SLO/2407/2006                  GQVLQGVLWLLLITGVQG 195
E1|KP313732.1|BVDV_1_Carlito                        GQMLQGILWLLLITGVQG 195
E1|FJ040215.1|BVDV_3_Th/04_KhonKaen                 GQIVQGVIWLLLITGAQG 195
E1|KC297709.1|BVDV_3_LVRI/cont-1                    GQIVQGVIWLLLITGAQG 195
E1|KC788748.1|BVDV_3_Italy-129/07                   GQIVQGVIWLLLITGAQG 195
E1|JX469119.1|BVDV_3_JS12/01                        GQIVQGVIWLLLITGAQG 195
E1|JX985409.1|BVDV_3_CH-KaHo/cont                   GQIVQGVIWLLLITGAQG 195
E1|AB871953.1|BVDV_3_D32/00_'HoBi'like              GQIVQGVIWLLLITGAQG 195
```

```
E1|HQ231763.1|BVDV_3_Italy-1/10-1                  GQIVQGVIWLLLITGAQG 195
E1|JQ612704.1|BVDV_3_Italy-83/10-ncp               GQIVQGVIWLLLITGAQG 195
E1|NC_018713.1|BVDV_3_LV03/12                      GQIVQGVIWLLLITGAQG 195
E1|KJ660072.1|Pestivirus_PG-2                      GQVVQGIVWLLLVTGAQG 194
E1|MH410816.1|Pestivirus_PG-2_GIRAFFE              GQVVQGIVWLLLVTGAQG 194
E1|AF144617.2|Pestivirus_giraffe-1_H138            GQVIQGIVWLLLVTGAQG 195
E1|KJ463422.1|BDV_FNK2012-1                        GQVVQGLVWLLLVTGAQG 195
E1|U70263.1|BDV_BD31                               GQVVQGLVWLLLVTGAKG 195
E1|KF925348.1|BDV_Coos_Bay-5_nc                    GQVVQGLVWLLLVTGAQG 195
E1|AB897785.1|BDV_X818                             GQVVQGLVWLLLVTGAQG 195
E1|AF144618.2|Pestivirus_reindeer-1_V60-Krefeld    GQIVQGLIWLLLVTGANG 195
E1|KC963426.1|BDV_JSLS12-01                        GQVVQGLLWLFLVTGAQG 195
E1|AF407339.1|BDV_Aveyron                          GQLIQGLIWLLLVTGAQG 195
E1|AY646427.1|CSFV_94.4/IL/94/TWN                  GQVVQGVIWLLLVTGAQG 195
E1|KP233070.1|CSFV_GXF29/2013                      GQVVQGVIWLLLVTGAQG 195
E1|KC851953.1|CSFV_IND/UK/LAL-290                  GQIVQGIIWLLLVTGAQG 195
E1|AF407339.1|CSFV_39                              GQVVQGIIWLLLVTGAQG 195
E1|FJ529205.1|CSFV_Zj0801                          GQVVQGIIWLLLVTGAQG 195
E1|KJ619377.1|CSFV_Bergen                          GQVVQGIIWLLLVTGAQG 195
E1|KU504339.1|CSFV_GD19/2011                       GQVVQGIIWLLLVTGAQG 195
E1|KM362426.1|CSFV_IND/AS/GHY/G4                   GQVVQGVIWLLLVTGAQG 195
E1|GQ923951.1|CSFV_SXCDK                           GQIVQGIIWLLLVTGAQG 195
E1|J04358.2|CSFV_Alfort/Tuebingen                  GQVVQGIIWLLLVTGAQG 195
E1|AY259122|CSFV_Alfort/Tuebingen                  GQVVQGIIWLLLVTGAQG 195
E1|KF669877.1|CSFV_JJ9811                          GQIVQGVIWLLLVTGAQG 195
E1|X87939.1|CSFV_Alfort/187                        GQIVQGVIWLLLVTGAQG 195
E1|X87939|CSFV_Alfort/187                          GQIVQGVIWLLLVTGAQG 195
E1|KJ660072.1|CSFV_Riems                           GQIVQGVVWLLLVTGAQG 195
E1|GU270877.1|BDV_H2121_Chamois-1                  GQIVQGLIWLLLVTGAQG 195
E1|KF918753.1|BDV_Gifhorn_genotype-3               GQVVQGLIWLLLVTGAQG 195
E1|JX428945.1|Pestivirus_Aydin/04-TR               GQVIQGIIWLLLVTGAQG 195
E1|AF037405.1|Pestivirus_Aydin/04-TR               GQVIQGIIWLLLVTGAQG 195
E1|KM408491.1|Pestivirus_Burdur/05-TR              GQVIQGAIWLLLVTGAQG 195
                                                   ** :*.   *::: ..:.
```

PLEASE NOTE: Showing colors on large alignments is slow.
